# Coupling spectral analysis and hidden Markov models for the segmentation of behavioural patterns

**DOI:** 10.1186/s40462-017-0111-3

**Published:** 2017-09-22

**Authors:** Karine Heerah, Mathieu Woillez, Ronan Fablet, François Garren, Stéphane Martin, Hélène De Pontual

**Affiliations:** 1 Ifremer, Sciences et Technologies Halieutiques, 10070, 29280 Plouzané, CS France; 2 IMT Atlantique, University Bretagne Loire, 29238 Brest, France

**Keywords:** Fourier transform, Non negative matrix factorization, Classification, Animal behaviour, European sea bass, Movement ecology, Diurnal and tidal cycles, Biologging, Data storage tags

## Abstract

**Background:**

Movement pattern variations are reflective of behavioural switches, likely associated with different life history traits in response to the animals’ abiotic and biotic environment. Detecting these can provide rich information on the underlying processes driving animal movement patterns. However, extracting these signals from movement time series, requires tools that objectively extract, describe and quantify these behaviours. The inference of behavioural modes from movement patterns has been mainly addressed through hidden Markov models. Until now, the metrics implemented in these models did not allow to characterize cyclic patterns directly from the raw time series. To address these challenges, we developed an approach to i) extract new metrics of cyclic behaviours and activity levels from a time-frequency analysis of movement time series, ii) implement the spectral signatures of these cyclic patterns and activity levels into a HMM framework to identify and classify latent behavioural states.

**Results:**

To illustrate our approach, we applied it to 40 high-resolution European sea bass depth time series. Our results showed that the fish had different activity regimes, which were also associated (or not) with the spectral signature of different environmental cycles. Tidal rhythms were observed when animals tended to be less active and dived shallower. Conversely, animals exhibited a diurnal behaviour when more active and deeper in the water column. The different behaviours were well defined and occurred at similar periods throughout the annual cycle amongst individuals, suggesting these behaviours are likely related to seasonal functional behaviours (e.g. feeding, migrating and spawning).

**Conclusions:**

The innovative aspects of our method lie within the combined use of powerful, but generic, mathematical tools (spectral analysis and hidden Markov Models) to extract complex behaviours from 1-D movement time series. It is fully automated which makes it suitable for analyzing large datasets. HMMs also offer the flexibility to include any additional variable in the segmentation process (e.g. environmental features, location coordinates). Thus, our method could be widely applied in the bio-logging community and contribute to prime issues in movement ecology (e.g. habitat requirements and selection, site fidelity and dispersal) that are crucial to inform mitigation, management and conservation strategies.

**Electronic supplementary material:**

The online version of this article (10.1186/s40462-017-0111-3) contains supplementary material, which is available to authorized users.

## Background

Animals exhibit a wide range of behaviours that have been learned and/or evolved to maximize fitness and reflect different activities such as resting, reproduction, migration, predation avoidance and foraging. These different behaviours/activities are adopted in suitable habitat (e.g. resource availabilities, physiologically suitable) that will ultimately result in an animal’s survival and successful reproduction [1]. However, wild animals can rarely be observed for more than a fraction of their daily activity. Consequently, our attempts to quantify behavioural patterns for modeling ecological processes often exclude cryptic, yet important behavioural events [2].

Over the last few decades, advances in bio-logging technologies have provided new insights into marine and terrestrial animals’ ecology by recording high resolution data for long periods of time, including their movements, physiology and reproductive biology, as well as concurrent environmental conditions [3]. Along with these technological advances, the field of movement ecology exploded because changes in movement patterns are the likely result of altered animal functional behaviour [2–4]. For instance, vertical movement patterns of marine pelagic species can be highly complex and reflect behaviours such as foraging, thermoregulatory excursions and spawning [5]. Movement ecology studies already provided crucial data (e.g. migration paths, foraging hotspots, site fidelity and dispersal, interactions with human activities) across taxa and realms to inform mitigation, management and conservation strategies [6–9]. However, optimizing the knowledge we can gain from animal movements on their biology and ecology requires quantitative tools to analyze these complex time series.

State-space models, especially hidden Markov models (HMM), have proven to be efficient in quantitatively detecting, segmenting and predicting behavioural patterns from movement data [4, 10–12]. They rely on the assumption that hidden behavioural modes correspond to different movement characteristics. For instance, HMM have been used: to distinguish between traveling versus foraging activities based on movement speed and sinuosity [10]; to detect spawning events from shovelnose sturgeon’s vertical movements [13]; to model flying activity of soaring raptor from acceleration data [11]. In most studies, the HMM applies directly to the raw movement data or simple descriptors such as instantaneous speed, local variance and distances [11, 14, 15]. As a result the model is mainly used to detect behavioural switches rather than focusing on the regularity and/or repetition of these changes.

Nonetheless, movement time series also often integrate cyclic patterns of animal’s behaviour and many have a periodicity equal to the ones of geophysical cycles (i.e. solar and lunar phases, season, year) they respond to [16]. These cycles induce spatio-temporal fluctuations in animals’ habitats by influencing their abiotic and biotic components (e.g. resource availability, physiological suitability, vulnerability to predators). In turn, animals’ distribution, activity levels and life history traits often reflect these geophysical cycles at different spatial and temporal scales. For instance, large marine mammals overtake seasonal migrations over thousands of kilometers between a winter reproductive site where there is less food available but where environmental conditions are suitable for the calf and a summer site where they forage actively [17]. Several species of fish have lunar and/or semi-lunar related spawning cycles both from a behavioural and physiological point of view [18]. At a smaller scale, zooplankton is known to conduct diel vertical migrations in the water column to avoid predation; while detecting such diurnal patterns in higher trophic levels provided information on their prey and foraging strategies [19, 20]. Detecting tidal and diel cycles in fish movement time series have also provided some information on their activity levels, position relative to the seafloor and spatial distribution [21, 22]. Obviously, the synchronizations of biological and behavioural activities with environmental cycles represent important adaptive strategies in animals to increase their reproductive success and resource acquisition as well as to decrease predation risks. Thus, detecting these patterns and the scale at which they occur from movement data contribute to our understanding on the ecology of species of interest in relation to their environment and ecosystem.

The identification of cyclic movement patterns can be difficult in a time series which results of a complex combination of signals that may confound each other. For instance, several cyclic behaviours could be simultaneously present in the time series along with non-periodic behaviours, spatio-temporal noises and outliers. In most studies, seasonal, diel, lunar and tidal rhythms were taken into account as qualitative variables, potentially included in statistical models, that are used to compare the observed patterns for different levels of the considered factor (e.g. day vs night, winter vs summer, tide levels) [7, 11, 23]. In comparison, relatively few ecological studies have investigated advanced time-frequency analyses (e.g, Fourier-based decompositions as well as wavelet analyses) to reveal cyclic vs. non-cyclic patterns [5, 22, 24]. However, to our knowledge, the interpretation of the derived time-frequency metrics remained mainly qualitative raising the need for further development to embed time-frequency metrics in state-of-the-art behavioural segmentation models (e.g. state-space and hidden Markov models, [4, 25, 26]).

In this study, we address this issue and develop a quantitative procedure for the characterization and segmentation of animal behaviour from 1-D movement data. Our contribution is two-fold: i) a generic approach for the extraction of metrics of cyclic behaviours and activity levels from a time-frequency analysis of 1-D movement time series, ii) the implementation of these spectral signatures into a HMM framework to identify and classify latent behavioural states along the time series. Simulated datasets were used to validate our approach which, was then applied to vertical movement data collected from wild European sea bass (*Dicentrarchus labrax*), a marine fish known to adapt its functional behaviour to diurnal and tidal cycles [27]. Previous studies also showed that sea bass tend to migrate between a coastal foraging ground in summer and a oceanic spawning ground in winter [28, 29]. We would expect that these different signals could be segregated one from the others and associated with different activities of the fish.

## Methods

All analyses were carried out using R. The code describing the whole procedure is provided in the Additional file 1 ﻿and a training dataset is provided in Additional file 2.

### Data storage tag data

Adult sea bass were internally tagged with Data Storage Tags (DSTs, CEFAS G5 long live) following the procedure described in [30]. Tagging operations were carried out in summer 2014 at Dunkirk (north-west of France, southern North Sea) and Saint Quay (north coast of Brittany, western English Channel); and in autumn 2014 at La Turballe (south coast of Brittany, northern Bay of Biscay) and Capbreton (south-west of France, southern Bay of Biscay) (Table 1). These sites are well separated along the French Atlantic coast and are associated with different environmental conditions.

**Table 1:** Summary of sea bass tagging locations and number of days spent at sea (mean ± standard deviation (sd))

Location			Day at sea			
Name	Latitude	Longitude	Mean ± sd	Min	Max	Total
Capbreton *N* = 10	43.640° N	1.449° W	350 ± 50	274	463	4005
Dunkirk N = 10	51.061 °N	2.368° E	402 ± 108	132	543	4022
La Turballe N = 10	47.346° N	2.516° W	368 ± 67	258	455	3676
Saint-Quay N = 10	48.656° N	2.826° W	350 ± 42	274	431	3502

Depth was recorded every 90 s. Long depth records (~ one year) for ten individuals per site were used in this study (Table 1). Each depth-time point in the dataset was attributed to a “day” or “night” factor for preliminary detection of diel cycles, and was also used to validate the model outputs. Having no prior knowledge on the fish locations, we used the sunrise time in western Ireland (12.55°W, 49.65°N) and the sunset time in eastern Denmark (7.93°E, 55.98°N) to delineate day vs night times, covering the widest area the fish could have gone to.

### Spectral analysis

#### Time-frequency analysis

Cyclic patterns and activity levels of sea bass vertical movements were first assessed using periodograms. They can be regarded as a representation of the amount of energy in a time series as a function of frequency [31]. On one hand, the activity level can be characterized by the overall magnitude of the signal. On the other hand, behaviours associated with cyclic movement patterns result in high-energy peaks in the periodogram; the frequency of these peaks being the characteristic frequency of the movement patterns (See Additional file 3: Figure S1 A for an illustration of this spectral characterization). When dealing with non-stationary time series, involving time-varying cyclic characteristics (e.g. tidal, diel and seasonnal cycles as well as different activity levels are confounded), as expected from movement time series, time-frequency analysis [31] resorts to the estimation of a time-varying periodogram.

**Fig. 1:**
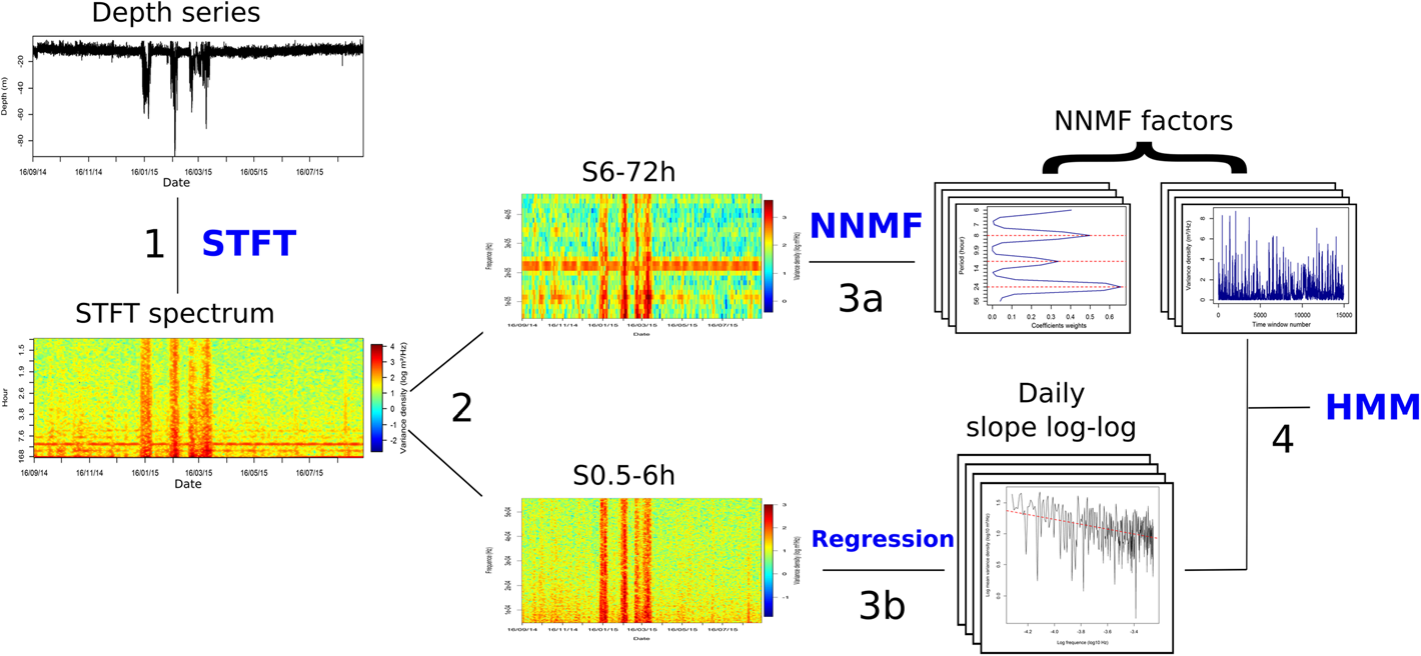
Sketch of the methodological procedure applied to raw depth time series: 1. the depth time series are analysed in the time-frequency domain using a Short Term Fourier Transform analysis in order to identify cyclic patterns and activity levels across time from periodograms; 2. The periodograms were divided into two parts: (i) between 6 and 72 h (S6-72 h); (ii) between half an hour and 6 h (S0.5-6 h); 3. For each STFT time window (i.e. one day) (a) the information contained in the 26 frequency bandwidths of S6-72 h was summarized by nine factors using a Non Negative Matrix Factorization (NNMF, see Additional file 3: Figure S3); (b) for the higher-frequency range S0.5-6 h, we computed an index of fine scale movement randomness by calculating the slope of the linear relationship between the log transformed variance densities and frequencies (see Additional file 3: Figure S1b); 4. We fitted HMMs to the time series of metrics formed by the nine-dimensional NNMF decomposition of each periodogram and the value of SLP_Log-Log_. Given a fitted HMM, we derive from each depth time series a time series of behavioural states (see Figs. 5 and 6)

**Fig. 2:**
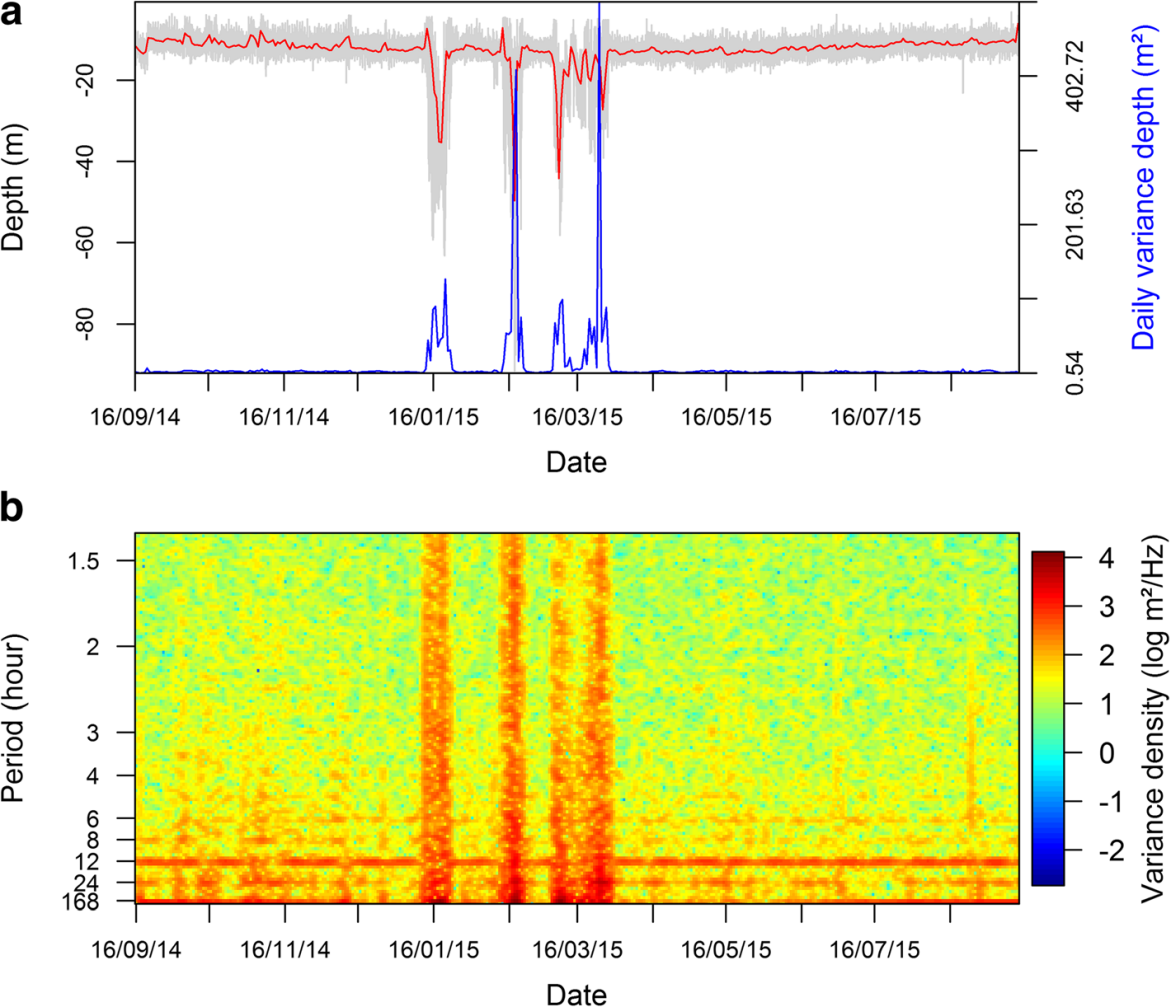
Example of **a** a raw depth time series (in grey) and associated daily median depth (in red) and depth variance (in blue) and **b** STFT-based (Short Term Fourier Transform) time-frequency analysis of the depth time series for an individual tagged at La Turballe (Tag # A11325)

Here, we applied a Short Term Fourier Transform (STFT, R package “e1071”, function *stft*, [32]) to each depth time series (Figs. 1 and 2). The STFT is a Fourier-based transform which provides information about the frequency content of local sections of a signal *s*(*t*) as it changes over time [33]:

$$ STFT\left[s(t)\right]\kern0.5em =\kern0.5em E\left(\tau, \kern0.5em \omega \right)\kern0.5em =\kern0.5em {\int}_{-\infty}^{+\infty}\kern0.5em s(t)\chi \left(t-\tau \right){e}^{-2\pi i\omega t} dt $$

The STFT can be regarded as the projection of the signal *s*(*t*) onto a set of base functions *χ*(*t* − *τ*)*e* ^−2π*iωt*^, *τ* and *ω* being respectively characteristic time and frequency of base functions. Note that this equation differs from the Fourier transform only by the presence of the window function *χ*. Here we considered a Hamming window [32] to fulfill local stationarity hypothesis. Practically, the STFT is generated by taking the Fourier transform of many time windows of the original signal shifted from one window to the next by a given time increment.

The STFT allows us to examine the evolution of the periodograms over time (Fig. 2b). It may be noted that STFT favors the time resolution over the spectral resolution. In particular, it does not resolve the spectral analysis for frequencies greater than the width of the considered window. In order to handle both fine scale vertical movements, as well as diurnal and tidal cycles, we applied a STFT with a 7 days window shifting by one-day increments (i.e. *days* 1 to 6, 2 to 7, 3 to 8, etc) (Figs. 1 and 2b). These settings are also consistent with the segmentation of behavioural patterns at a daily resolution.

#### Segregation of the STFT periodogram according to movement pattern scales

The resulting STFT periodograms (Fig. 2b) displayed strong modes and higher energies between 72 and 6 h (lower frequencies; e.g. daily movements, tidal and diel cycles) while it was more homogeneous and associated to lower values between 6 and 0.5 h (highest frequencies; i.e. fine-scale and random movements). We expect these two frequency ranges to potentially relate to different behavioural and environmental processes, which may explain the differences in the exhibited energy levels. To avoid hiding small scale movements (high-frequency component) by the daily scale movements (low-frequency component), we isolated the two frequency ranges: (1) between half an hour and 6 h (S0.5-6 h, 309 frequency bandwidths); (2) between 6 and 72 h (S6-72 h, 26 frequency bandwidths) (Fig. 1, Additional file 3: Figure S1).

#### Dimension reduction: Calculation of an index of randomness and non negative matrix factorization

In order to ensure a balanced analysis between the two frequency ranges and to decrease the number of variables included in our classification scheme, we applied a dimension reduction strategy to each frequency range as follows.

For fine scale behaviours, S0.5-6 h, we calculated the slope of the log-log relationship between the energies and frequencies (hereafter “Slp_Log-log_”, Fig. 1 and Additional file 3: Figure S1B):

$$ \log \left(E\left(\tau \kern0.5em =\kern0.5em t,\omega \right)\right)\kern0.5em =\kern0.5em {A}_t\kern0.5em \log \left(\omega \right)\kern0.5em +\kern0.5em {B}_t $$

The slope is a good indicator of activity levels and randomness of the movements. While uncorrelated noise processes (i.e. random movements) correspond to a slope of 0; correlated random processes are associated with a negative slope (i.e. directed vertical movements in the water column) [34], with greater slope (more negative) corresponding to longer-scale dependencies. These relationships are features of Matèrn processes, a family of classical Gaussian processes whose spectral density is asymptotically described by power laws. For example, an asymptotic slope of −1 corresponds to a one-dimensional Ornstein-Uhlenbeck process, a first-order auto-regressive model characterized by an exponential covariance [21].

Daily movement patterns and cycles (S6-72 h), were still represented by spectral energies for 26 frequencies per time window (on average 3735 ± 126 time windows per site). First, these spectral values were normalized for each frequency of the periodogram for all the individuals and sites pooled together (i.e. each column of the whole S6-72 h matrix, 26 × 14,939). This ensures variance homogeneity among frequency bandwidths, sites and individuals. We then applied a dimension reduction method to S6-72 h (Fig. 1). Rather than the classical Principal Component Analysis, we apply a Non Negative Matrix Factorization (NNMF) analysis. The NNMF analysis is commonly used in signal processing (e.g. image compression, image and sound recognition, text classification; [35, 36]) and is more appropriate for datasets with only positive values, such as spectral energies. More specifically, here, the extracted basis factors (equivalent to the principal components of the PCA) can truly be interpreted as spectral patterns with non-negative values. NNMF factorizes a matrix *A* (n time windows (*τ*) x n frequency bandwidths (*ω*)) into two rank-*k* matrices *W*(*τ* × *k*) and *H*(*k* × *ω*), such that *A* is the most accurately approximated by *WH* and *k* is inferior to rank(*A*) ([36] and references therein).

We applied a NNMF analysis (R package “NMF”, function *nmf*, [36]) to S6-72 h for all individuals and sites pooled together, such that the whole dataset is summarized by the same NNMF factors before classification (Fig. 1). More specifically, we used the Alternating Least Square (ALS) algorithm as it was computationally faster than other approaches [37] for similar results. To determine the optimal number of factorization ranks (*k*) we ran the NNMF from two to 20 factors and computed quality measures of the results ([36] and references therein). Several quality and performance measures (e.g. cophenetic coefficients and RSS (Residual Sum of Squares)) have been proposed to choose the optimal *k* value. As suggested by [38, 39] we chose the *k* value for which, the cophenetic correlation coefficients (which indicate the dispersion of the consensus matrix) decreased afterward and for which the RSS (Residual Sum of Squares) curve presented an inflexion point (Additional file 3: Figure S2). Accordingly, the best approximation of S6-72 h was obtained with nine NNMF factors (Additional file 3: Figure S2 and S3). Each factor is associated with different frequency peaks (Additional file 3: Figure S3 #a) and their corresponding occurrence along the time series (Additional file 3: Figure S3 #b).

### Segmentation of latent behavioural states using hidden Markov models

Hidden Markov models (HMM) are widely acknowledged as powerful tools for modelling and classifying animal behaviours, while simultaneously dealing with inherent auto-correlation and noise of movement time series [4, 11]. Detailed mathematical descriptions of HMMs and broader state-space models may be found in previous publications (e.g. [25, 26]). We only outline the general framework hereafter.

A HMM is a stochastic time series involving two layers: an observable state-dependent process and an unobservable state process. In the context of animal behaviour, a HMM assumes that an observation *O* at a particular time step (e.g. location, distance travelled, speed) results from a distribution (also called observation distribution) associated with a behavioural state *S*. The time series of these hidden behavioural states is modelled as a first-order Markov chain. Along that chain, the probabilities of switching from one state to the others are determined by a transition matrix. The probability of a behavioural state j at time t only depends on the state at time t-1, and the transition probabilities to state j at time t [4, 11].

#### HMM parameterization and implementation

Let us denote by *S* = {*S*_*t*_} the latent behavioural states series to be inferred at a daily resolution, and *O* = {*O*_*t*_} = {*W*_*t*_, *A*_*t*_} the observation series of the coefficients of the nine retained NNMF factors (*W*_*t*_, Additional file 3: Figure S3 #b) and the SLP_Log-Log_ slope values (*A*_*t*_, Additional file 3: Figure S1 B). The latent variables *S*_1_ , …*S*_*T*_ represent the hidden states of some underlying mechanism that generated the observed data. For *S*_*t*_ = *s*, we assume that the distribution *P*(*O*_*t*_| *S*_*t*_ = *s*) follows a multivariate Gaussian distribution with a diagonal covariance structure to make model inference easier and numerically more stable. Experiments were carried out to test different distributions (R package “depmixS4”, functions “*depmix*” and “*fit*”, [40]), the multivariate Gaussian being the most adequate for our dataset.

Regarding the transition probabilities, we used individuals as a covariate on the transition matrix to consider individual heterogeneity in switching dynamics. Let us denote by *z*_*t*_ the covariates representing the individual at time *t*. The transition probability is then parameterized using a multinomial logit model as follows:

$$ P\left({S}_{t+1\kern0.5em }=\kern0.5em j|{S}_t\kern0.5em =\kern0.5em i,{z}_t\right)\kern0.5em =\kern0.5em {p}_{ij}^{(t)}\left({z}_t\right)\kern0.5em =\kern0.5em \frac{e^{\left({\beta}_O^{(ij)}+{Z}_t{\beta}_1^{(ij)}\right)}}{\varSigma_{k=1}^M{e}^{\left({\beta}_O^{(ik)}+{z}_t{\beta}_1^{(ik)}\right)}},\kern0.5em for\kern0.5em i,j=1,\dots \kern0.5em M\kern0.5em states $$

Each row of the transition matrix is parameterized by a baseline category logistic multinomial, meaning that the parameter for the base category is fixed at zero. The default baseline category is the first state. This means that all individuals share the same observation models but involves individual-specific transition matrices (e.g. ${p}_{ij}^{(t)}\left({z}_t\kern0.5em =\kern0.5em \mathrm{A}10639\right)$ for individual A10639). For a given number of behavioural states, HMM calibration was carried out according to a Maximum Likelihood criterion using an expectation-maximization algorithm (EM) (R package “depmixS4”, functions “*depmix*” and “*fit*”, [40]). It resorts to the concatenation of all individual time series into a single time series with the associated covariate time series. Given the estimated HMM parameters, we proceeded with the analysis of individual movement patterns and used the Viterbi algorithm to compute the most likely sequence of behavioural states [40].

#### Model selection

Choosing the optimal number of states in a HMM is a critical issue [4, 11]. This is particularly true in behavioural ecology when no prior knowledge on quantitative metrics to describe animal behaviours are available [11]. The use of information criteria (e.g. Akaike Information Criterion, AIC; Bayesian Information Criterion, BIC) solely for model selection is controversial. For instance, the use of AIC only in HMM selection tends to favour overly complex models which can make ecological interpretations of estimated states difficult [11]. Besides, the use of the Integrated Completed Likelihood (ICL), which is a variant to the BIC, has proven to be efficient in HMM selection ([41] and references therein). Model selection based on the BIC minimization is a common approach as it includes both model estimation negative log-likelihood and penalties on its complexity (See BIC equations in [42]). The ICL index is equal to the BIC penalized by the mean entropy of the posterior probabilities of the estimated model (See equations in [40]). This entropy penalizes clustering configurations exhibiting overlapping states. It means that models with lower entropy are associated with better separated states and will be favoured. Thus, due to the extra penalization term, the ICL tends to be less prone to discriminate overlapping states, essentially becoming an efficient model-based criterion that can be used to outline the clustering structure in the data [41]. Finally, we chose the optimal number of states (between 3 and 10, see Additional file 3: Figure S4) for our dataset by retrieving the best compromise between the ICL, entropy and the least complex model in order to facilitate ecological interpretations (Additional file 3: Figure S5).

### Simulation-based validation of the approach

To assess the performance of our approach, we designed a ground truthed simulation-based experiment as follow. The simulated dataset involves three depth time series with a 90-s resolution over 366 days. Three behavioural states were included in these simulations. In addition, we reproduced individual variability, by considering different transition matrices for each state time-series. For each individual, the states time series were sampled from the individual transition matrix. Then, the simulation of the depth D over time t was conditional to behavioral states S, and was made of two components: an autoregressive process AR and a periodic signal SW (Eq. 1), the parameters of which are detailed in Additional file 3: Tables S1 and S2.

1 $$ D\left(t|S=i\right)\kern0.5em =\kern0.5em \left({\alpha}_i AR\left(t,{\theta}_i^{AR}\right)+{\beta}_i\right)\kern0.5em +\kern0.5em \left({\gamma}_i SW\left(t,{\theta}_i^{SW}\right)+{\delta}_i\right) $$

For state 1 and 2, the movement followed a cyclic pattern of 24 h and 12.8 h respectively (Additional file 3: Table S1), associated with a Gaussian random walk with an autoregressive process (Additional file 3: Table S2). For state 3, the movement was characterized by a lognormal random walk with an autoregressive process (to mimic sea bass deeper dives, Additional file 3: Table S2). Additional file 3: Figure S6 A, C, E illustrate these simulated state time-series. We then, applied the whole procedure to these datasets, including model selection using the ICL index and cross-validated the estimated states to the simulated ones using confusion matrices.

## Results

### Simulation study

The mean normalized periodogram of each behavioural state for the three-state HMM showed that behavioural states from our simulation-based experiment were discriminated according to their activity levels and spectral signatures (State 1: peaks at 24 and 8 h (harmonic of the characteristic frequency), State 2: peaks at 12.8 h, State 3: no peak) within 6 to 72 h (Additional file 3: Figure S7). The proposed HMM succeeded in correctly estimating the mean characteristics of the behavioural states and reached an overall mean accuracy of 94% for the segmentation of the hidden states from the depth series (Additional file 3: Table S3).

### General features

The procedure we developed (Fig. 1) was applied to the DST depth time series of 10 sea bass per four independent sites along the French Atlantic coast. For each individual, depth was recorded every 90 s for a year (on average) providing a total of 3502 to 4022 days at sea at each site for our analyses (Table 1). The similarity in dataset sizes between sites ensures that the analyses are homogeneously driven by all sites.

### Detection of rhythmicity from spectral analysis

The STFT analysis (performed on each time series) highlighted, over time, the strongest changes in an individual’s activity levels in the water column (e.g. highest depth variations on 16/01/15, Fig. 2). In addition, the STFT analysis identified patterns within the low and high-frequency bandwidths of the periodogram, which were not indicated by changes in the median depth and/or depth variance (Fig. 2). Firstly, the mean periodogram calculated from the STFT for the low frequencies bandwidths (S6-72 h) displayed strong peaks at 24, 12.8, 12 and 8 h highlighting the occurrence of cyclic patterns in individuals’daily behaviour (Additional file 3: Figure S1A). These peaks correspond to the spectral signatures of two geophysical cycles: the diurnal cycle (peaks at 24, 12 and 8 h, with the second and third ones being harmonics [i.e. echoes] of the 24 h peak) and the tidal cycle (peak at 12.8 – semi-diurnal tide component). Secondly, for the high-frequency range (S0.5-6 h), the Slp_Log-log_ values indicate that individuals’ small scale movements are directed as they depict a clear autocorrelation (Additional file 3: Figure S1B, −0.7 ± 0.2 for all individuals and day pooled together).

### Behavioural classification

#### Model outputs

HMMs were fitted using the coefficients of the nine NNMF factors and the SLP_Log-Log_ values as daily observations, and individuals as covariates for the transition matrix. Different number of states were tested from 3 to 10 (Additional file 3: Figure S4). According to the ICL criterion, the optimal number of states was seven (Additional file 3: Figure S5). However, in order to facilitate ecological interpretation, a less complex model characterized by five states was retained. Indeed, the seven states-model only differ from the five states-model by doubling the two states corresponding to the fish being the least active (Additional file 3: Figure S4C and E).

Then, the behavioural state associated with daily observations was re-assigned to the periodogram and Slp_Log-log_ matrix and to the time series for all individuals according to the corresponding date. The activity level can be characterized by the overall magnitude of the signal. In addition, behaviours associated with cyclic movement patterns result in high-energy peaks in the periodogram; the frequency of these peaks being the characteristic frequency of the movement patterns.

**Fig. 3:**
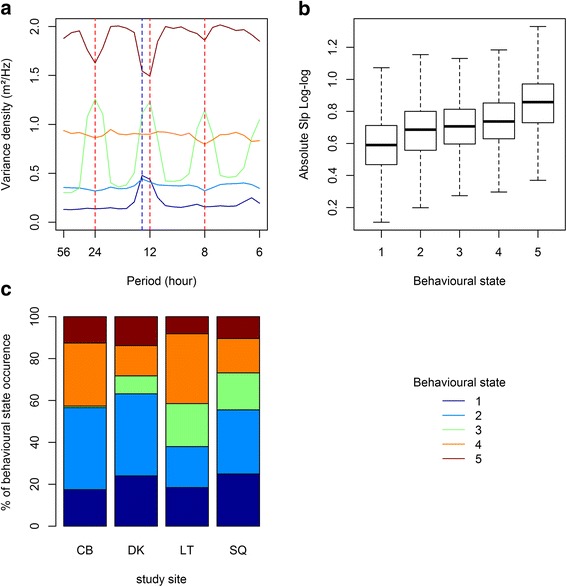
Mean normalized periodogram of each behavioural state for the low-frequency range (6 to 72 h) (**a**); box-plot of SLP_Log-Log_ values (median is indicated in bold) for each behavioural state (**b**); and relative occurrence frequencies of the behavioural states at each site (**c**), discriminated from the fitted five-state HMM. In (**a**) and (**b**), results are presented for all sites and individuals pooled together. CB: Cap Breton, DK: Dunkirk, LT: La Turballe, SQ: Saint-Quay

The mean normalized periodogram of each behavioural state for the retained HMM showed that behavioural states were discriminated according to their activity levels and spectral signature (i.e. the occurrence of peaks) within 6 to 72 h (Fig. 3a). Despite, the Slp_Log-log_ values showing that fine scale movements (between 0.5 and 6 h) were directed (Additional file 3: Table S5), they did not seem to account for much in discriminating behavioural states (Fig. 3b). Behavioural state one (*St1*), two (*St2*) and five (*St5*) occurred in relatively similar proportions among sites (Fig. 3c). Conversely, the proportions of behavioural state three (*St3*) and four (*St4*) varied more between sites, and *St3* was almost not adopted by individuals from Capbreton (Fig. 3c). This likely reflects different behavioural adaptations according to regional differences in abiotic and biotic conditions.

#### Activity levels and spectral signature of the different behavioural classes

**Fig. 4:**
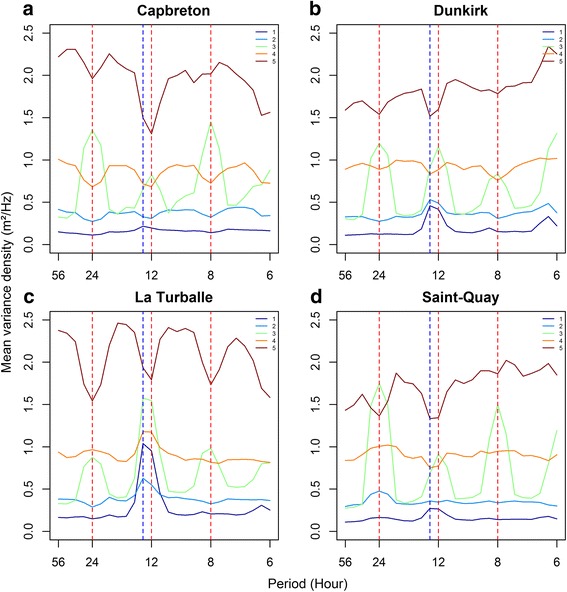
Spectral signature and activity levels associated to each behavioural states of the fitted five-state HMM for all individuals pooled together at each site. The orange and blue dotted lines indicate the diurnal and tidal periodicities, respectively. **a** Capbreton. **b** Dunkirk. **c** La Turballe. **d** Saint-Quay

Fish were the least active while displaying behavioural state one (*St*1, 0.22 ± 0.17 m^2^/Hz), followed by *St*2 (*St*1, 0.43 ± 0.32 m^2^/Hz), *St3* (0.77 ± 0.55 m^2^/Hz), *St*4 (*St*4, 1 ± 0.60 m^2^/Hz) and *St*5 (*St*2, 2 ± 0.61 m^2^/Hz) (Figs. 3a and 4). *St*1 was also characterized by a strong tide signal, while *St*2 mean energy density was generally homogeneous across frequency bandwidths (Fig. 3a). The same patterns were observed among sites, although the magnitude of the tide signal varied between sites and was also present in *St*2 at Dunkirk (Fig. 4b) and La Turballe (Fig. 4c). Fish displayed a strong diurnal behaviour in *St*3 and this pattern was consistent among sites even though the magnitude of the diurnal peaks varied between them (Figs. 3a and 4). The spectral signature of *St*4 was homogeneous among frequency bandwidth, showing that fish did not adopt strong cyclic movement patterns in this behavioural state (Fig. 3a). The patterns observed for *St*3 and *St*4 were consistent among sites (Fig. 4), except at La Turballe where there was also a tidal signal (Fig. 4c). For *St*5, the energy was minimal at 24, 12.8, 12 and 8 h, revealing no cyclic pattern and/or an inverted diurnal pattern (Fig. 3a). In addition, the stronger variability of spectral features associated with *St*5 among sites compared to the other behavioural states (Fig. 4), suggested that *St*5 corresponds to fish adopting more complex behaviours.

#### Depth specific periodic behaviour: Diurnal and tidal rhythms

**Fig. 5:**
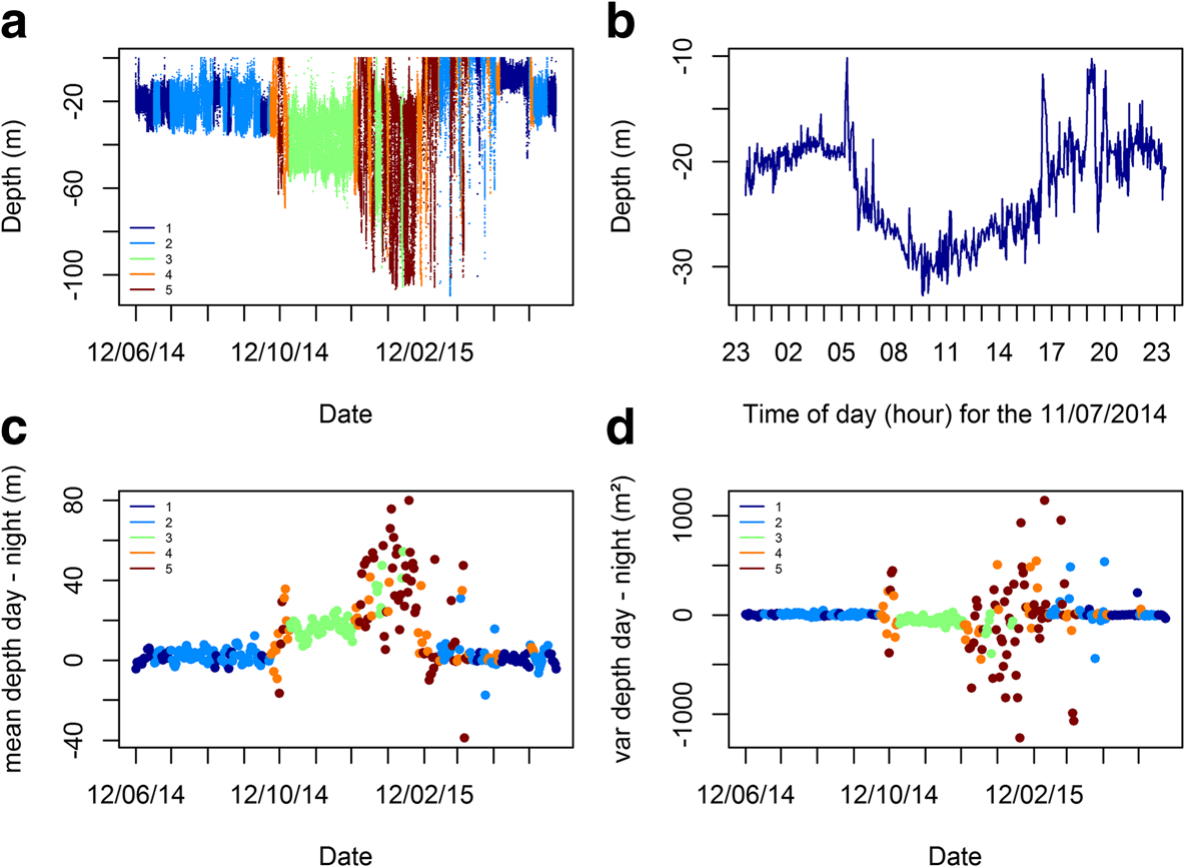
The different behavioural states inferred with the fitted five-state HMM are represented along the depth time series (**a**). The tidal signature associated to state *St*1 (**b**) and diurnal rythms (**c**-**d**) associated to state *St*3 and *St*5 are also represented. The differences in daily mean depth (**c**) and variability (**d**) between day and night are representative of diurnal changes in the position in the water column and activity levels, respectively. *St*2 and *St*4 represent intermediate activity levels and are not characterized by a tidal signature nor a clear diurnal rhythm, respectively. The behavioural analysis is reported for one individual tagged at Saint-Quay (Tag # A10639)

In order to confirm the tidal and diurnal spectral signatures observed in the mean normalized periodograms we looked at the depth series of the corresponding behavioural states. As such, the tide signal clearly exhibited by *St*1 periodogram was also observable in the time series (see Fig. 5b). Similarly, *St*3 and *St*5 were associated with the highest differences in depth ranges and variations between day and night (Fig. 5c-d, Additional file 3: Table S4). More specifically, it seems that *St*3 corresponded to periods when the individuals displayed a directed diurnal activity such as diving deeper during the day but being equally active during the day or at night. In contrast, *St*5 corresponded to less clear patterns in day or night depth occupancies, and more variable activity levels between day and night (Fig. 5c-d, Additional file 3: Table S4). Conversely, *St*1 and *St*2 were generally associated with the lowest differences in depth ranges and variations between day and night (Fig. 5d-e, Additional file 3: Table S4).

#### Depth series and behavioural classes

**Fig. 6:**
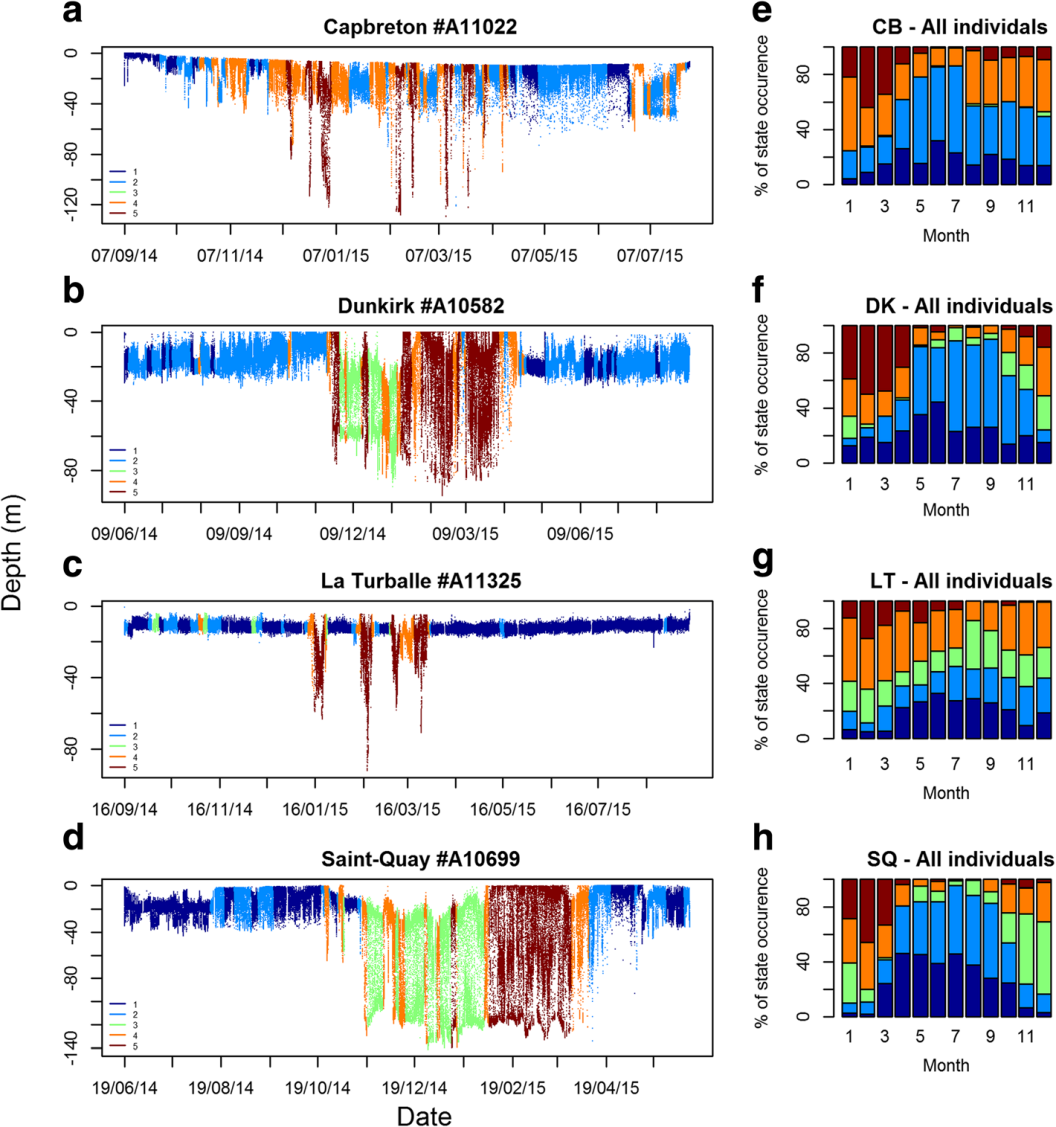
Segmented behavioural states represented along the depth time series of one individual at each site (**a**, **b**, **c**, **d**). The segmentation exploited the retained fitted five-state HMM; as a result, all states are not necessarily displayed by all individuals. The monthly percentage of the behavioural state occurrences are also represented for each site (**e**, **f**, **g**, **h**)

Similar to activity levels and diurnal patterns, *St*1 and *St2* were generally associated with the shallower depth ranges and variations, followed by *St*4; *St*3 and *St*5 which corresponded to the deepest positions in the water column and largest depth variations (Figs. 5 and 6, Additional file 3: Table S4). However, there were inter-site differences between depth ranges and variations associated with each behavioural state. In addition, not all individuals always displayed all the behavioural states during their time at sea (Fig. 6).

The occurrences of the different behavioural states were temporally well-defined and appeared at similar times throughout the annual cycle (Fig. 6). At Capbreton, Dunkirk and Saint-Quay (Fig. 6e–h), the fish were the least active in summer during the feeding season (main occurrence of *St*1 and *st*2) while they were the most active in winter during the breeding season (main occurrence of *St*5). At these sites the fish mainly adopted *St*4 (intermediate activity level, no cyclic behaviours) from September–October to April–May. At Dunkirk and Saint-Quay, diurnal movements (*St*3) mainly occurred from September to January just before and at the beginning of *St*5 occurrence. At La Turballe, behaviours occurred at similar times, but the patterns were less pronounced than at the other sites which likely result from a larger proportion of fish being residents in that area (data not presented).

## Discussion

Movement pattern variations are reflective of behavioural switches, and are likely associated with different life history traits in response to the animals’ abiotic and biotic environment. Detecting these different behaviours, the scale and periodicities at which they occur and their switches can provide rich information on the underlying processes driving these movement patterns. The extraction of such information from movement time series requires tools that objectively describe and quantify these behaviours. The innovative aspects of our method lie within the combined use of powerful mathematical tools (spectral analysis and hidden Markov models) to identify and then classify behavioural states. We were able to discriminate between these behaviours by deciphering movement cyclic patterns and activity levels from a 1-D movement time series. In the current trend, where bio-logging technologies (and thus movement ecology studies) are increasing rapidly, our method could be widely applied to any species and customized to answer a broad range of ecological questions.

### Methodological discussion

Our method combines the use of a time-frequency analysis (STFT) and a dimension reduction analysis (NNMF). These techniques accurately extracted and summarized the key metrics of different movement patterns (i.e. cyclic behaviour and activity levels) contained within the time series. These metrics were then implemented in a Markovian model framework, used as a classification tool, to identify sea bass vertical behaviours. The whole procedure is fully automated which makes it applicable to large high-resolution datasets.

Time-frequency analyses in ecology have been mainly used for analyzing acoustic signals (e.g. [43, 44]). Nonetheless, a few studies applied time-frequency techniques to detect cyclic behaviours in terrestrial and marine vertebrate such as diurnal, tidal, as well as semi-lunar and lunar cycles [5, 22, 24, 45, 46]. These analyses are well suited to analyzing and extracting complex information confounded in long-term high-resolution datasets such as those from archival tagging studies. Periodic, non-periodic behaviours and different activity regimes can be extracted directly from animal movements without requiring other indices (e.g. variance as an index of activity, time of day, seasons) or additional environmental metrics (e.g. day vs night for diel patterns, [23, 47]; ebb and flood for tidal ones, [11]). This is particularly useful for 1-D time series, when neither measures of in situ light levels, nor animals’ position, are recorded by the tags. Conversely, more classical approaches (i.e. [48]) using depth mean or median (indicative of fish distribution in the water column) and variance (indicative of fish activity regime) did not allow to segregate states associated with the same level activity but associated with different behavioural cycles (figure not presented; see also [49]). For instance, when the fish were intermediately active, we distinguished two states, with and without diurnal cycles whereas using classical metrics only identify one state. In addition, using the raw depth series and/or descriptive metrics of fish position in the water column result into a classification biased toward depth values. Furthermore, a statistical analysis combining dive metrics and direct use of diel, tidal state covariates or time of day, would implicitly assume predefined priors on the timing of the behavioural states as well as their spectral characteristics (i.e. cyclic patterns), which may hardly be defined if not misleading or inaccurate. Polansky et al. [24] also illustrated the strength of using time-frequency analyses in combination with correlated random walk models to detect the periodicity and scales at which spatial movements and activities occur [24].

Identifying the timing and extent of behavioural patterns along a movement time series is not feasible with classical Fourier transform or autocorrelation function. For instance, in Shepard et al. [5] and Scott et al. [22], the authors identified the overall occurrences of periodic patterns, but had no automated processes for isolating them along the time series. They had to perform supplementary analyses and subsample the times-series (e.g. every month in [5] or day in [22]) to extract this information over time. In order to overcome this limitation, we used a time-frequency analysis, namely the STFT, which allowed us to analyze potential time-varying vertical movement patterns. Our setting (Hamming window, seven days by one day increments) enabled us to identify cyclic patterns that repeat over a week. However, the simulation experiments showed that the HMM was less accurate in inferring the appropriate state at the transition between two states. This is likely due to a loss of time resolution inherent to the STFT window we chose. In addition, our setting does not permit the extraction of larger periodic patterns, such as seasonal ones. For instance, Scott et al. [22] identified putative spawning behaviour of Pacific Halibut at a scale of 6–10 days as well as lunar and semi-lunar periodic behaviour over several weeks. This said, any frequency range could be examined depending of the process one wants to highlight, and the users need to adapt the size of the STFT window according to their study question. Obviously, the lower the considered frequency ranges are, the lower the time resolution of detected behavioural shifts will be.

Identifying and quantifying behavioural switches using the outputs of time-frequency analyses is another issue. As discussed in Polansky et al. (2010) [24], ecological interpretation in the time-frequency domain is not always straightforward. They may also result in numerous variables (here, the number of frequency bandwidths, e.g. 335 in our study) which may be difficult to directly use in a classification framework. The practitioner could focus on pre-defined frequency ranges of interest if behavioural patterns are known a priori. However, this precludes from discovering new patterns of individuals’ movements.

With this in mind, we optimized the classification process by summarizing the information (i.e. dimension reduction) of the STFT analysis by using a NNMF. It provided a lower-dimensional representation of the periodograms while still accounting for significant movement information. While we finally retain the optimal number of NNMF factors (i.e. according to the RSS and cophenetic coefficients), supplementary experiments revealed that increasing or decreasing (from 3 to 20) the number of NNMF factors implemented in the HMMs did not change significantly the behavioural states discriminated. It shows that our approach is not sensitive to the NNMF, which nonetheless seems to be important to speed up the inference and avoid numerical pitfalls (i.e. which occurred when considering the raw STFT data for the entire datasets).

Hidden Markov models are particularly well suited for analyzing an animal’s movement time series because they directly account for the fact that any corresponding information will be driven by the underlying behavioural state or general activity level of the animal [4, 11]. In addition, HMMs deal with the strong auto-correlation inherent to any time series in a mechanistic way, by allowing states to be persistent over time rather than omitting the feature completely (e.g. cluster analyses, [50]) or including it in an error term (e.g. Generalized Mixed Effect Models, [51]). This feature is also crucial in our procedure as Fourier-based descriptors involve long-term (low-frequency) and short-term (high-frequency) correlations.

In behavioural ecology, HMMs can be used in a supervised approach to identify pre-defined behavioural states of interest ([52]; e.g. [53]), or in a unsupervised approach (e.g. the one we described). While the unsupervised approach offers the opportunity to learn about unknown behaviours of an animal [4, 11], it also have some limitations. Within an unsupervised framework, the determination of the number of states results from some trade-off between model complexity, likelihood and behavioural plausibility [4, 11]. The ecological interpretation of the latent behaviours relies on expert knowledge of the biology and ecology of the species of interest and is made a posteriori [11, 54]. For instance, in this study, given that actual fish behaviours at sea cannot be observed, direct behavioural state validation, from an ecological point of view, (e.g. [53, 55]) could not be performed. Nevertheless, simulated non-stationary time series with periodic patterns and our results revealed the efficiency of HMMs, combined with a time-frequency analysis, in discriminating behavioural shifts. In our application to sea bass depth time series, behavioural states were well-defined and persistent over time, also providing support for the proposed framework. The inter-site similarity in energy levels and spectral signatures associated with the different states stressed the robustness of our method in characterizing and segmenting similar patterns in animals’ behaviour along movement time series.

### Behavioural mode inferences

By applying our approach to European sea bass depth time series data, we showed that these animals occupy different parts of the water column, adopt different activity regimes and their vertical movements could be associated with environmental cycles. In addition, the timing of the different behaviours throughout the annual cycle amongst individuals suggest these behaviours are likely related to seasonal functional behaviours such as feeding, migrating and spawning (Fig. 6). However, little is known about the species ecology in its natural environment or its role in the marine ecosystems [27–29] and as such, the behavioural inference we can make are limited and must be taken cautiously.

The tidal signature associated with *St*1 and *St*2 is observed as a consequence of the fish being the least active in these states and likely corresponds to the water height above fish varying with tide. Consequently, the presence/absence of a tidal signal could provide information on the vertical and spatial location of the fish [21, 22]. For instance, the presence of a tide signal, in combination with an inactive behaviour, likely corresponds to the fish remaining inactive close to the seafloor. Alternatively, its absence could be linked to the spatial location of the fish (e.g. La Turballe: strong tidal signal, vs Capbreton: low tidal signal), or indicate that the fish are active horizontally, rather than vertically, and behaved in response to sea surface, rather than seafloor conditions [5, 22]. The fact that these behaviours mainly occurred during summer (i.e. seabass feeding season, [27]) may suggest that *St*1 and *St*2 could be related to foraging activities (i.e. feeding, digestion, “sit and wait” hunting strategy). Fish most active behaviours (*St*3 and *St*5) were also associated with diurnal and diurnal-inverted signals and mostly occurred in winter (i.e. seabass spawning season, [27]). Such behaviours could be adopted to favour reproductive success in response to their environment, such as predator avoidance, and physiological constraints, but also food uptake before energetically demanding spawning events and/or thermoregulatory excursions. As for *St*4, it could be described as a non-periodic behaviour with intermediate activity levels, and could correspond to the fish travelling between areas [22, 27].

In this study, individuals and sites were pooled together in order to extract a set of behaviours that would be overall representative of the population as well as comparable between sites and individuals. Inter-individual variations and transition matrices were not investigated in this study and would deserve a study on its own. In theory, one could choose to fit the HMM per individual and perform some post-fitting analyses to study inter-individual/site variations. While it would increase the overall complexity of the model, it would also decrease the amount of data available for the inference of the HMM parameters, with potential overfitting risks. Furthermore, it might results in behavioural states that would not be comparable between individuals, especially if working on a large number of them. Thus, we recommend to apply procedures that are as integrative as possible, such as the approach proposed here or hierarchical modelling (e.g. [12]). The application of our method to a larger dataset (i.e. more individuals at multiple sites over a longer time frame), as well as the thorough examination of state transitions statistics, would provide useful insights into the seasonal movement patterns of sea bass and their underlying drivers, such as temperature [27].

### Method applications and perspectives

Experts in bio-logging technologies and movement ecology, in concert with conservation agencies, have identified key questions and goals that are applicable to terrestrial and marine species [3]. In this framework, the method we developed should contribute to the understanding of animal habitat requirements and selection, and their interactions with the ecosystem.

First, while GPS and Argos locations are available for air-breathing marine animals (e.g. reptiles, marine mammals, birds), geolocations from animals that remain below the surface (i.e. fish) are achieved by an animal-borne logger, and later used to reconstruct animal movement. In several geolocation models light, temperature, depth and tidal signals have been used to locate a posteriori the animals [21, 30]. Our analysis strongly suggested that some behavioural states (*St*1 & *St2*) relate to tide signals, which in turn could be used as tide-driven cues for geolocation issues (e.g. [21]). Furthermore, our model provided information on the vertical position of the fish in the water column and their level of activity. This may offer key information that help disentangle functional behaviours and its links with the three-dimensional movement of animals (e.g. [15, 56]). Such behaviour-driven complementary cues could be integrated in geolocation models to constraint displacement parameters and refine locations’ estimation (see [21, 30]).

Second, assessing how environmental features shape animal movement is essential for two main reasons: (i) provide insights into the drivers of behavioural changes, thus improving our knowledge of species biology and ecology; and (ii) a better understanding of species habitat requirements. Both are crucial for assessing how climate change and anthropogenic activities will impact individuals and populations. HMMs have great potential for investigating the links between animal behaviour and their environment by using an integrative approach (e.g. [11, 57]). In particular, HMMs offer the flexibility to include (1) several observation variables, such as a set of behavioural observations as well as combined behavioural and environmental variables; and (2) any covariates that could influence the probability of behavioural switches (e.g. environment, individual variability; [4, 11, 15, 58]).

## Conclusions

Despite improved bio-logging technologies and the proliferation of movement ecology studies, there remains a need for generic quantitative tools for extracting information from increasingly large bio-logging datasets. The method we present here successfully enabled to identify and classify individual behaviours, taking into account, in an integrative and quantitative manner, both movement activity levels and cyclic patterns, directly from a 1-dimensional movement time series. This method relies on powerful, but generic, mathematical tools that can be customized to any type of time series dataset and species. This broadens its applicability to animal movement studies that aim to investigate major ecological questions.

## Additional files

Additional file 1: Algorithm of the method (training dataset available in additional file 2). (R 13 kb)

Additional file 2: Training dataset for one tagged seabass. (CSV 2933 kb)

Additional file 3: Table S1. Parameter values conditional to behavioral states for the autoregressive process component of the simulated depth time series. **Table S2.** Parameter values conditional to behavioral states for the periodic signal component of the simulated depth time series. **Table S3.** Confusion matrix for cross-validation between the simulated known states and the HMM estimated states. **Table S4.** Depth ranges for each 5 behavioural states. **Table S5.** Mean values and standard deviations of NNMF factors and Slp_Log-Log_ variables per HMM states. **Figure S1.** Spectral signature and activity levels of movements between 6 and 72 h ( orange and blue dotted lines indicate diurnal and tidal periodicities, respectively), averaged over time (A); and an index of movements randomness and activity levels (B). Individual #A11325 tagged at La Turballe. **Figure S2.** The optimal number of factorization ranks (red dotted line) of the NNMF analysis based on the cophenetic coefficient (A) and the RSS curve (B). **Figure S3.** NNMF outputs obtained fromperiodograms between 6 and 72 h for all individuals and sites pooled together. Periodograms associated with each factor of the selected 9-dimensional NNMF (#a). Coefficients time series of the NNMF decomposition of the daily periodograms (#b). **Figure S4.** Mean normalized periodogram (S6-72 h) associated with each behavioural state inferred from HMM ran with three (A) to ten (H) latent states. **Figure S5.** Model selection criterions: BIC (A), model entropy (B) and ICL (C). The red dotted line indicates the five-states HMM we retained. **Figure S6.** Known (A, C, E) and estimated (B, D, F) behavioural states for three simulated individual series with different state switching dynamics (Table S1). **Figure S7.** Spectral signature and activity levels associated to each behavioural states of the fitted three-state HMM for all simulated individuals pooled together. The orange and blue dotted lines indicate diurnal and tidal periodicities, respectively. (DOCX 1349 kb)

## Abbreviations

CB: Capbreton
DK: Dunkirk
DST: Data Storage Tag
EM: Expectation-maximization algorithm
HMM: Hidden Markov Model
ICL: Integrated Completed Likelihood index
LT: La Turballe
NNMF: Non Negative Matrix Factorization
PCA: Principal Component Analysis
RSS: Residual Sum of Squares
S0.5-6 h: Periodogram for the frequencies corresponding to 0.5 to 6 h periods
S6-72 h: Periodogram for the frequencies corresponding to 6 to 72 h periods
Slp_Log-log_: Slope of the log-log relationship between the energies and frequencies
SQ: Saint-Quay
*St1 to 5*: Hidden Markov Model behavioural state 1 to 5
STFT: hort Term Fourier Transform

## References

[1] Stevick PT, McConnell BJ, Hammond PS. Patterns of Movement. In Hoelzel AR, editor, Marine Mammal Biology: an evolutionary approach. Blackwell. 2002. p. 185-216.

[2] Brown DD, Kays R, Wikelski M, Wilson R, Klimley AP (2013). Observing the unwatchable through acceleration logging of animal behavior. Anim Biotelemetry.

[3] Hays GC, Ferreira LC, Sequeira AMM, Meekan MG, Duarte CM, Bailey H (2016). Key questions in marine Megafauna movement ecology. Trends Ecol Evol.

[4] Phillips JS, Patterson TA, Leroy B, Pilling GM, Nicol SJ (2015). Objective classification of latent behavioral states in bio-logging data using multivariate-normal hidden Markov models. Ecol Appl.

[5] Shepard EL, Ahmed MZ, Southall EJ, Witt MJ, Metcalfe JD, Sims DW (2006). Diel and tidal rhythms in diving behaviour of pelagic sharks identified by signal processing of archival tagging data. Mar Ecol Prog Ser.

[6] Douglas-Hamilton I, Krink T, Vollrath F (2005). Movements and corridors of African elephants in relation to protected areas. Naturwissenschaften.

[7] Meyer CG, Papastamatiou YP, Holland KN (2007). Seasonal, diel, and tidal movements of green jobfish (Aprion Virescens, Lutjanidae) at remote Hawaiian atolls: implications for marine protected area design. Mar Biol.

[8] Trebilco R, Gales R, Baker GB, Terauds A, Sumner MD (2008). At sea movement of Macquarie Island giant petrels: relationships with marine protected areas and regional fisheries management organisations. Biol Conserv.

[9] Hindell MA, Lea M-A, Bost C-A, Charrassin J-B, Gales N, Goldsworthy S, et al. Foraging habitats of top predators, and areas of ecological significance, on the Kerguelen Plateau. Kerguelen Plateau Mar. Ecosyst. Fish. Abbeville Fr. Soc. Francaise Ichtyologie. 2011:203–15.

[10] Jonsen ID, Basson M, Bestley S, Bravington MV, Patterson TA, Pedersen MW (2013). State-space models for bio-loggers: a methodological road map. Fourth Int Symp Bio-Logging Sci.

[11] Leos-Barajas V, Photopoulou T, Langrock R, Patterson TA, Watanabe YY, Murgatroyd M (2017). Analysis of animal accelerometer data using hidden Markov models. Methods Ecol Evol.

[12] Leos-Barajas V, Gangloff E, Adam T, Langrock R, van Beest FM, Nabe-Nielsen J (2017). Multi-scale modeling of animal movement and general behavior data using hidden Markov models with hierarchical structures.

[13] Holan SH, Davis GM, Wildhaber ML, DeLonay AJ, Papoulias DM (2009). Hierarchical Bayesian Markov switching models with application to predicting spawning success of shovelnose sturgeon. J R Stat Soc Ser C Appl Stat.

[14] Patterson TA, Basson M, Bravington MV, Gunn JS (2009). Classifying movement behaviour in relation to environmental conditions using hidden Markov models. J Anim Ecol.

[15] Bestley S, Jonsen ID, Hindell MA, Harcourt RG, Gales NJ (2015). Taking animal tracking to new depths: synthesizing horizontal–vertical movement relationships for four marine predators. Ecology.

[16] Li Z, Han J, Ding B, Kays R (2012). Mining periodic behaviors of object movements for animal and biological sustainability studies. Data Min Knowl Discov.

[17] Lockyer C, Brown S (1981). The migration of whales.

[18] Takemura A, Rahman MS, Park YJ (2010). External and internal controls of lunar-related reproductive rhythms in fishes. J Fish Biol.

[19] Fuiman LA, Davis R, Williams T (2002). Behavior of midwater fishes under the Antarctic ice: observations by a predator. Mar Biol.

[20] Hays GC. A review of the adaptive significance and ecosystem consequences of zooplankton diel vertical migrations. Migr. Dispersal Mar. Org. Spring. 2003:163–70.

[21] Pedersen MW, Righton D, Thygesen UH, Andersen KH, Madsen H (2008). Geolocation of North Sea cod (Gadus Morhua) using hidden Markov models and behavioural switching. Can J Fish Aquat Sci.

[22] Scott JD, Courtney MB, Farrugia TJ, Nielsen JK, Seitz AC (2016). An approach to describe depth-specific periodic behavior in Pacific halibut (Hippoglossus Stenolepis). J Sea Res.

[23] Heerah K, Andrews-Goff V, Williams G, Sultan E, Hindell M, Patterson T (2013). Ecology of Weddell seals during winter: influence of environmental parameters on their foraging behaviour. Deep Sea res. Part II top. Stud. Oceanography.

[24] Polansky L, Wittemyer G, Cross PC, Tambling CJ, Getz WM (2010). From moonlight to movement and synchronized randomness: Fourier and wavelet analyses of animal location time series data. Ecology.

[25] Patterson T, Thomas L, Wilcox C, Ovaskainen O, Matthiopoulos J (2008). State–space models of individual animal movement. Trends Ecol Evol.

[26] Jonsen ID, Myers RA, Flemming JM (2003). Meta-analysis of animal movement using state-space models. Ecology.

[27] Vázquez FJS (2014). Muñoz-Cueto JA.

[28] Pawson M, Pickett G, Kelley D (1987). The distribution and migrations of bass, Dicentrarchus Labrax L., in waters around England and Wales as shown by tagging. J. Mar. biol. Assoc. U. K.

[29] Pickett G, Pawson M (1995). Sea bass: biology, exploitation and conservation. Oceanogr Lit Rev.

[30] Woillez M, Fablet R, Ngo T-T, Lalire M, Lazure P, de Pontual H (2016). A HMM-based model to geolocate pelagic fish from high-resolution individual temperature and depth histories: European sea bass as a case study. Ecol Model.

[31] Flandrin P. Time-frequency/time-scale analysis: Academic press; 1998.

[32] Meyer D, Dimitriadou E, Hornik K, Weingessel A, Leisch F. e1071: Misc Functions of the Department of Statistics (e1071), TU Wien. R package version 1.6–3. 2014;

[33] Sejdić E, Djurović I, Jiang J (2009). Time--frequency feature representation using energy concentration: an overview of recent advances. Digit Signal Process.

[34] Rasmussen CE, Williams CKI (2006). Covariance functions.

[35] Lee DD, Seung HS (1999). Learning the parts of objects by non-negative matrix factorization. Nature.

[36] Gaujoux R, Seoighe C (2010). A flexible R package for nonnegative matrix factorization. BMC Bioinformatics.

[37] Kim H, Park H (2007). Sparse non-negative matrix factorizations via alternating non-negativity-constrained least squares for microarray data analysis. Bioinformatics.

[38] Brunet J-P, Tamayo P, Golub TR, Mesirov JP (2004). Metagenes and molecular pattern discovery using matrix factorization. Proc Natl Acad Sci.

[39] Hutchins LN, Murphy SM, Singh P, Graber JH (2008). Position-dependent motif characterization using non-negative matrix factorization. Bioinformatics.

[40] Visser I, Speekenbrink M (2010). depmixS4: An R-package for hidden Markov models. J Stat Softw.

[41] Bertoletti M, Friel N, Rastelli R (2015). Choosing the number of clusters in a finite mixture model using an exact integrated completed likelihood criterion. Metro.

[42] Robles B, Avila M, Duculty F, Vrignat P, Begot S, Kratz F. Methods to choose the best Hidden Markov Model topology for improving maintenance policy. 2012. p. 1.

[43] Richard G, Vacquie-Garcia J, Jouma’a J, Picard B, Genin A, Arnould JPY (2014). Variation in body condition during the post-moult foraging trip of southern elephant seals and its consequences on diving behaviour. J Exp Biol.

[44] Wisniewska DM, Johnson M, Teilmann J, Rojano-Doñate L, Shearer J, Sveegaard S (2016). Ultra-high foraging rates of harbor porpoises make them vulnerable to anthropogenic disturbance. Curr Biol.

[45] Hartill B, Morrison M, Smith M, Boubee J, Parsons D (2004). Diurnal and tidal movements of snapper (Pagrus Auratus, Sparidae) in an estuarine environment. Mar Freshw Res.

[46] Graham RT, Roberts CM, Smart JC (2006). Diving behaviour of whale sharks in relation to a predictable food pulse. J R Soc Interface.

[47] Bestley S, Gunn JS, Hindell MA (2009). Plasticity in vertical behaviour of migrating juvenile southern bluefin tuna ( *Thunnus maccoyii* ) in relation to oceanography of the south Indian Ocean. Fish Oceanogr.

[48] Langrock R, King R, Matthiopoulos J, Thomas L, Fortin D, Morales JM (2012). Flexible and practical modeling of animal telemetry data: hidden Markov models and extensions. Ecology.

[49] Pinto C, Spezia L (2015). Markov switching autoregressive models for interpreting vertical movement data with application to an endangered marine apex predator.

[50] Dragon A-C, Bar-Hen A, Monestiez P, Guinet C (2012). Horizontal and vertical movements as predictors of foraging success in a marine predator. Mar Ecol Prog Ser.

[51] Zuur A, Ieno EN, Walker N, Saveliev AA, Smith GM. Mixed effects models and extensions in ecology with R: New York: Springer; 2009.

[52] Hastie T, Tibshirani R, Wainwright M. Statistical learning with sparsity: the lasso and generalizations: CRC Press; 2015.

[53] Joo R, Bertrand S, Tam J, Fablet R (2013). Hidden Markov models: the best models for forager movements?. PLoS One.

[54] Gloaguen P, Mahévas S, Rivot E, Woillez M, Guitton J, Vermard Y (2015). An autoregressive model to describe fishing vessel movement and activity. Environmetrics.

[55] Hijmans RJ (2012). Cross-validation of species distribution models: removing spatial sorting bias and calibration with a null model. Ecology.

[56] Heerah K, Hindell M, Andrew-Goff V, Field I, McMahon CR, Charrassin J (2017). Contrasting behavior between two populations of an ice-obligate predator in East Antarctica. Ecol Evol.

[57] Bestley S, Patterson TA, Hindell MA, Gunn JS (2010). Predicting feeding success in a migratory predator: integrating telemetry, environment, and modeling techniques. Ecology.

[58] Bestley S, Jonsen ID, Hindell MA, Guinet C, Charrassin J-B (2012). Integrative modelling of animal movement: incorporating in situ habitat and behavioural information for a migratory marine predator. Proc R Soc B Biol Sci.

