# Supplementary material for: Coupling spectral analysis and hidden Markov models for the segmentation of behavioural patterns
Source: Mov Ecol. 2017 Sep 22;5:20. doi: 10.1186/s40462-017-0111-3 (PMC5609058; doi:10.1186/s40462-017-0111-3)
Supplement: Supplementary file 3 — Parameter values conditional to behavioral states for the autoregressive process component of the simulated depth time series. Table S2. Parameter values conditional to behavioral states for the periodic signal component of the simulated depth time series. Table S3. Confusion matrix for cross-validation between the simulated known states and the HMM estimated states. Table S4. Depth ranges for each 5 behavioural states. Table S5. Mean values and standard deviations of NNMF factors and SlpLog-Log variables per HMM states. Figure S1. Spectral signature and activity levels of movements between 6 and 72 h ( orange and blue dotted lines indicate diurnal and tidal periodicities, respectively), averaged over time (A); and an index of movements randomness and activity levels (B). Individual #A11325 tagged at La Turballe. Figure S2. The optimal number of factorization ranks (red dotted line) of the NNMF analysis based on the cophenetic coefficient (A) and the RSS curve (B). Figure S3. NNMF outputs obtained fromperiodograms between 6 and 72 h for all individuals and sites pooled together. Periodograms associated with each factor of the selected 9-dimensional NNMF (#a). Coefficients time series of the NNMF decomposition of the daily periodograms (#b). Figure S4. Mean normalized periodogram (S6-72 h) associated with each behavioural state inferred from HMM ran with three (A) to ten (H) latent states. Figure S5. Model selection criterions: BIC (A), model entropy (B) and ICL (C). The red dotted line indicates the five-states HMM we retained. Figure S6. Known (A, C, E) and estimated (B, D, F) behavioural states for three simulated individual series with different state switching dynamics (Table S1). Figure S7. Spectral signature and activity levels associated to each behavioural states of the fitted three-state HMM for all simulated individuals pooled together. The orange and blue dotted lines indicate diurnal and tidal periodicities, respectively. (DOCX 1349 kb) [file 40462_2017_111_MOESM3_ESM.docx]

**Supplementary information**

**Table S1.** Parameter values conditional to behavioral states for the autoregressive process component of the simulated depth time series.

| **States** | **Autoregressive process** | | | | | | |
| --- | --- | --- | --- | --- | --- | --- | --- |
|  | order | autoregressive component | innovation mean | innovation std dev. | innovation distribution | coefficient α | intercept β |
| 1 | 1 | 0.7 | 0 | 2.5 | normal | 1 | -10 |
| 2 | 1 | 0.9 | 0 | 0.5 | normal | 1 | -10 |
| 3 | 1 | 0.8 | 0 | 2.0 | lognormal | -1 | 15 |

**Table S2.** Parameter values conditional to behavioral states for the periodic signal component of the simulated depth time series.

| **States** | **Periodic signal** | | | |
| --- | --- | --- | --- | --- |
|  | characteristic frequency | wave form | Coefficient γ | intercept δ |
| 1 | 24h | square | 20 | -20 |
| 2 | 12.8h | sine | 10 | 0 |
| 3 | none | none | none | none |

**Table S3.** Confusion matrix for cross-validation between the simulated known states and the estimated states with our approach and retained three states HMM.

|  | **Simulated known states** | | |
| --- | --- | --- | --- |
| **Estimated HMM states** | **1** | **2** | **3** |
| **1** | 410211 | 670 | 0 |
| **2** | 4217 | 257863 | 0 |
| **3** | 39459 | 14261 | 304362 |
| **Balanced accuracy** | 0.95 | 0.97 | 0.96 |
| **Sensitivity** | 0.90 | 0.95 | 1 |
| **Specificity** | 0.99 | 0.99 | 0.93 |

**Table S4.** Depth ranges for each behavioural classes issued from the five states HMM according to sites and time of the day. CB: Cap Breton, DK: Dunkirk, LT: La Turballe, SQ: Saint Quay.

| **Depth per site per HMM state (m, mean ± sd)** | | | | | | |
| --- | --- | --- | --- | --- | --- | --- |
|  | **Time of the day** | **1** | **2** | **3** | **4** | **5** |
| **CB** | All | 8 ± 7 | 11 ± 8 | 18 ± 20 | 14 ± 12 | 24 ± 28 |
|  | Day | 9 ± 7 | 12 ± 9 | 27 ± 25 | 16 ± 13 | 34 ± 34 |
|  | Night | 7 ± 7 | 10 ± 8 | 12 ± 13 | 12 ± 10 | 18 ± 21 |
| **DK** | All | 14 ± 8 | 16 ± 9 | 30 ± 19 | 20 ± 19 | 31 ± 31 |
|  | Day | 15 ± 8 | 17 ± 9 | 37 ± 20 | 25 ± 21 | 38 ± 33 |
|  | Night | 12 ± 8 | 14 ± 9 | 26 ± 16 | 17 ± 18 | 27 ± 29 |
| **LT** | All | 13 ± 4 | 14 ± 5 | 12 ± 3 | 12 ± 5 | 17 ± 15 |
|  | Day | 13 ± 4 | 15 ± 5 | 13 ± 3 | 13 ± 5 | 18 ± 16 |
|  | Night | 13 ± 4 | 14 ± 6 | 11 ± 3 | 11 ± 6 | 16 ± 15 |
| **SQ** | All | 14 ± 10 | 17 ± 13 | 51 ± 38 | 35 ± 33 | 44 ± 44 |
|  | Day | 17 ± 10 | 20 ± 13 | 67 ± 43 | 42 ± 36 | 56 ± 45 |
|  | Night | 12 ± 9 | 13 ± 13 | 43 ± 32 | 31 ± 30 | 37 ± 42 |
| **All sites** | All | 13 ± 8 | 14 ± 10 | 30 ± 30 | 18 ± 19 | 29 ± 33 |
|  | Day | 14 ± 9 | 16 ± 9 | 35 ± 35 | 20 ± 20 | 36 ± 30 |
|  | Night | 11 ± 8 | 13 ± 10 | 26 ± 26 | 16 ± 17 | 25 ± 30 |

**Table S5.** Estimated mean values and standard deviations for the nine NNMF factors and Slp_Log-Log_ variables issued from the fitted five state HMM.

|  | **NNMF factor 1** | **NNMF factor 2** | **NNMF factor 3** | **NNMF factor 4** | **NNMF factor 5** | **NNMF factor 6** | **NNMF factor 7** | **NNMF factor 8** | **NNMF factor 9** | **Slp Log-Log** |
| --- | --- | --- | --- | --- | --- | --- | --- | --- | --- | --- |
| **State 1** | 0.15 ± 0.14 | 0.09 ± 0.08 | 0.08 ± 0.09 | 0.07 ± 0.08 | 0.09 ± 0.09 | 0.12 ± 0.10 | 0.09 ± 0.11 | 0.44 ± 0.46 | 0.08 ± 0.08 | -0.58 ± 0.20 |
| **State 2** | 0.23 ± 0.21 | 0.24 ± 0.20 | 0.24 ± 0.20 | 0.25 ± 0.24 | 0.20 ± 0.18 | 0.30 ± 0.24 | 0.22 ± 0.22 | 0.31 ± 0.26 | 0.23 ± 0.20 | -0.68 ± 0.19 |
| **State 3** | 0.56 ± 0.50 | 0.24 ± 0.24 | 0.17 ± 0.20 | 0.10 ± 0.14 | 0.80 ± 0.70 | 0.26 ± 0.26 | 1.19 ± 1.02 | 0.94 ± 0.78 | 0.15 ± 0.19 | -0.71 ± 0.17 |
| **State 4** | 0.48 ± 0.43 | 0.56 ± 0.47 | 0.59 ± 0.48 | 0.68 ± 0.60 | 0.47 ± 0.43 | 0.64 ± 0.53 | 0.64 ± 0.59 | 0.59 ± 0.57 | 0.65 ± 0.51 | -0.74 ± 0.18 |
| **State 5** | 1.21 ± 1.20 | 1.24 ± 1.28 | 1.32 ± 1.22 | 1.36 ± 1.50 | 1.21 ± 1.10 | 1.42 ± 1.34 | 1.16 ± 1.20 | 0.83 ± 0.89 | 1.48 ± 1.38 | -0.85 ± 0.18 |

**
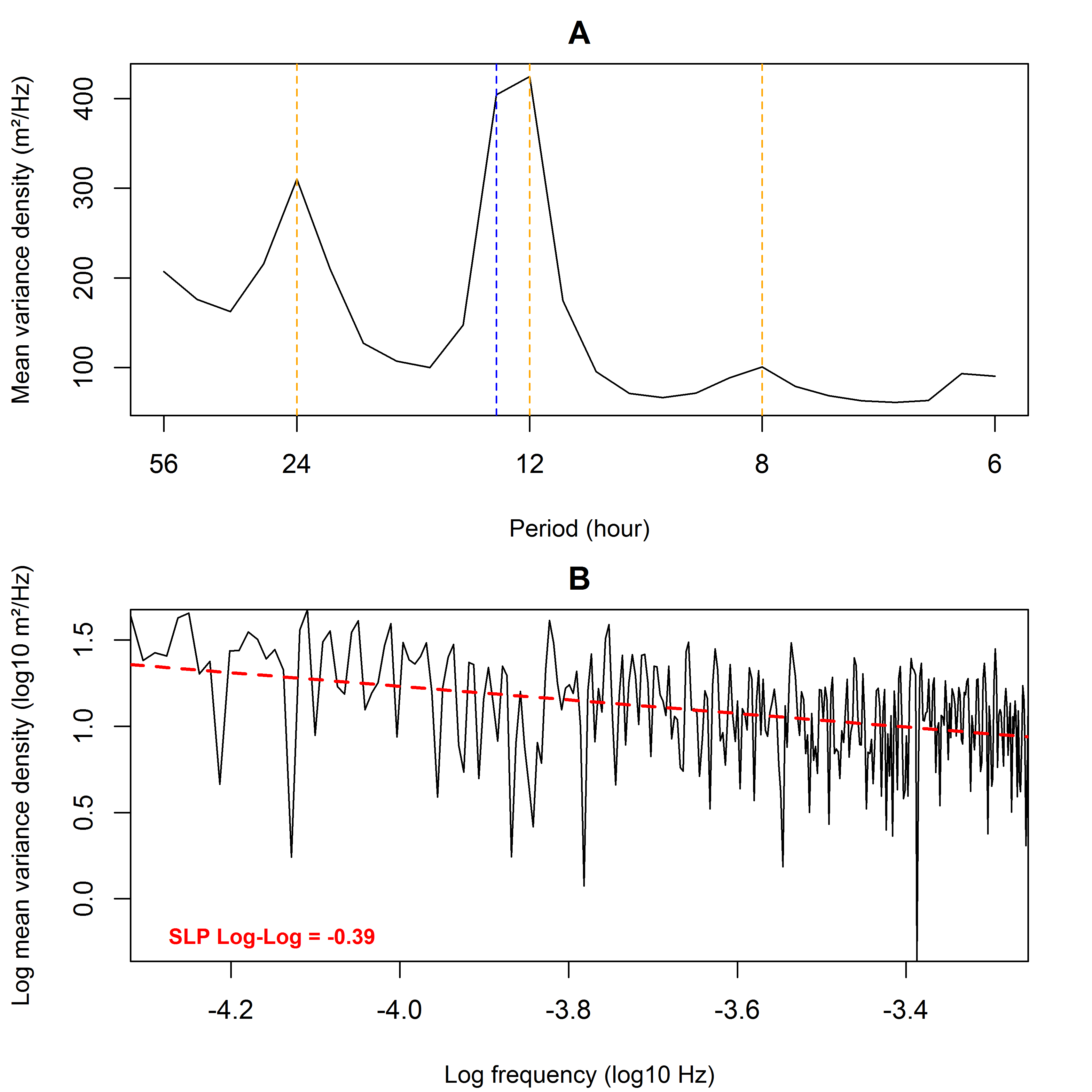
**

**Figure S1.** Spectral signature and activity levels of sea bass movements between 6 and 72 hours (S6-72h, orange and blue dotted lines indicate the diurnal and tidal periodicities, respectively), averaged over time (A); and an index of movements randomness and activity levels: the the regression slope in log-log scale (Slp_Log-log_) calculated for S0.5-6h, represented here for one day (*i.e.* one time window of the STFT analysis) (B) (See Material and Methods section “spectral analysis”). The two parts of the spectrum are represented for individual #A11325 tagged at La Turballe.


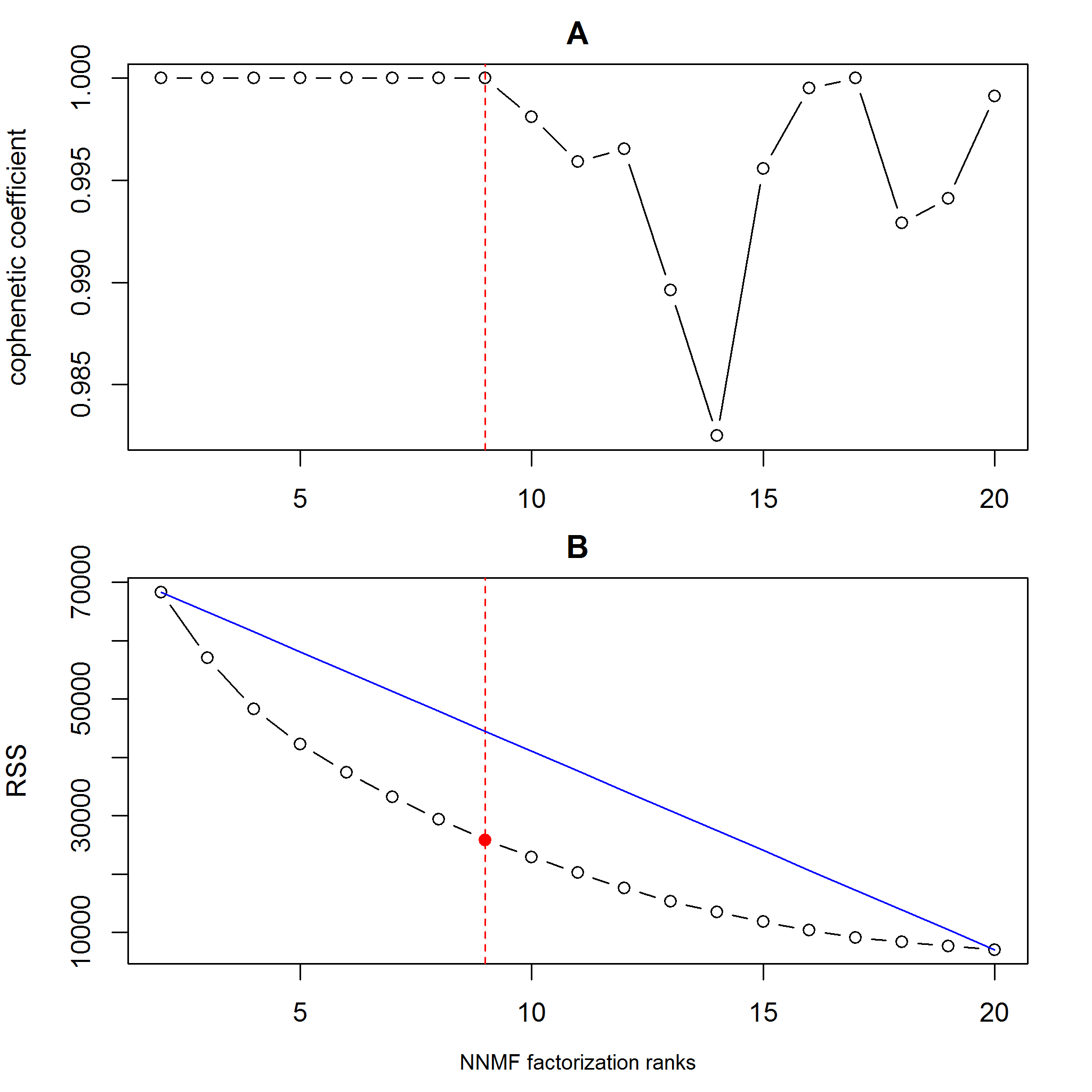


**Figure S2.** The optimal number of factorization ranks (red dotted line) of the Non Negative Matrix Factorization analysis corresponded to the first value after which the cophenetic coefficient started to decrease (A) and to the inflexion point (red dot) of the RSS (Residual Sum of Squares) curve (B). The inflexion point corresponded to the maximal distance between the RSS curve and its linear approximation between the first and last RSS values.


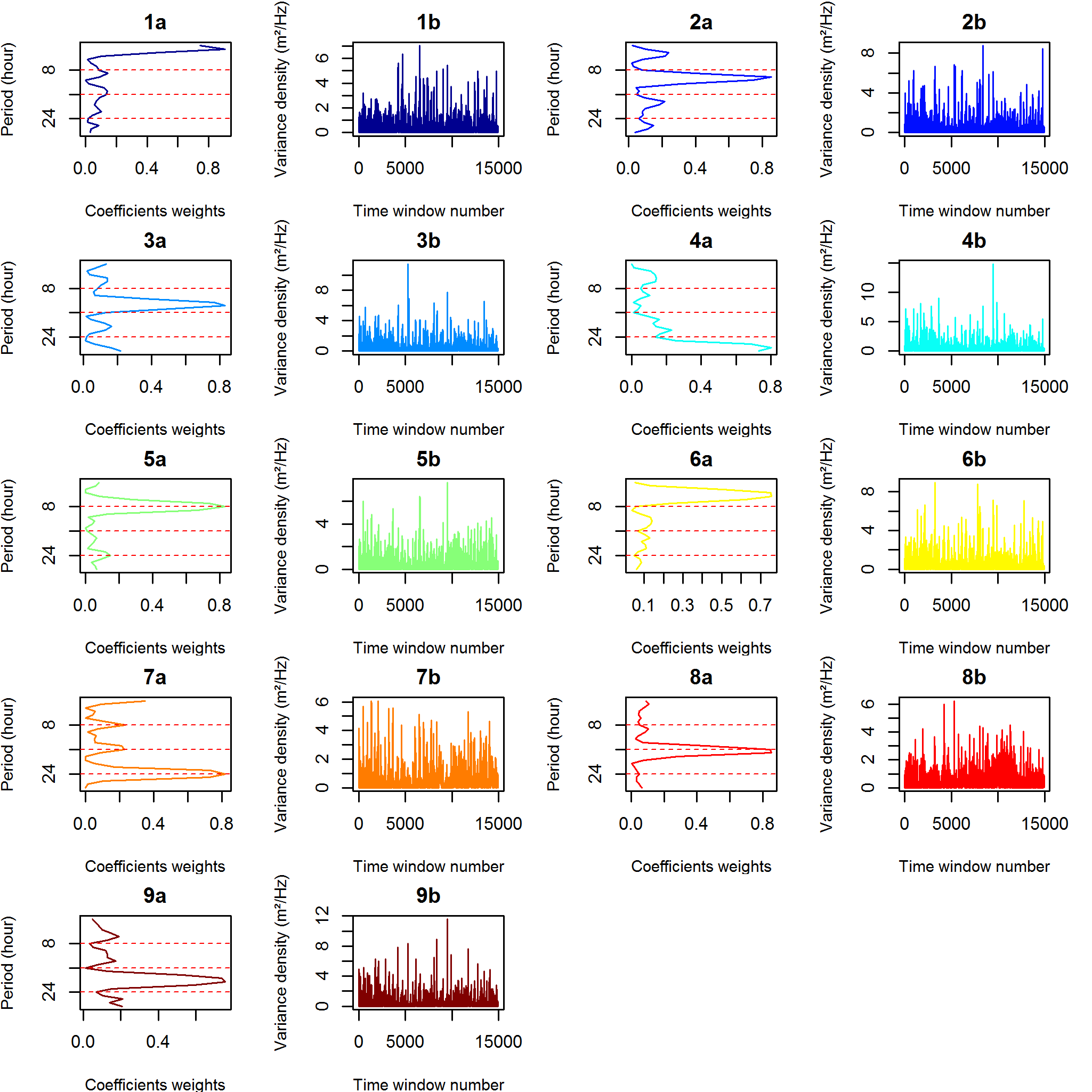


**Figure S3.** Illustration of the NNMF applied to the periodograms between 6 and 72 hours for all individuals and sites pooled together. Periodogram associated with each factor of the selected 9-dimensional NNMF (#a). Time series of the coefficients of the NNMF decomposition of the daily periodograms (#b) (See Material and Methods section “Non Negative Matrix Factorization).


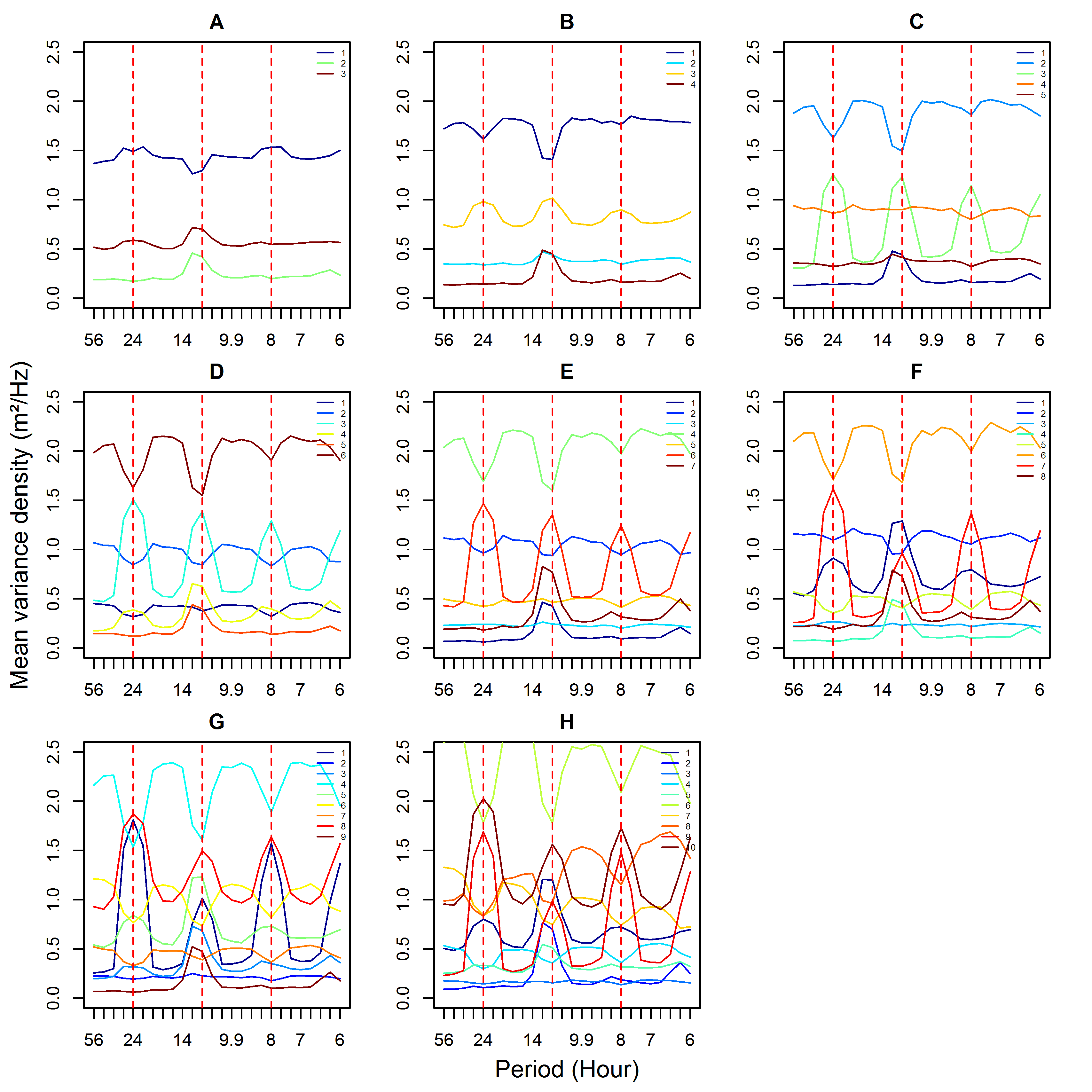


**Figure S4.** Mean normalized periodogram (S6-72h) associated with each behavioural state inferred from a set of nine Hidden Markov Models ran to discriminate from three (A) to ten (H) latent states (see Appendix S4).

**
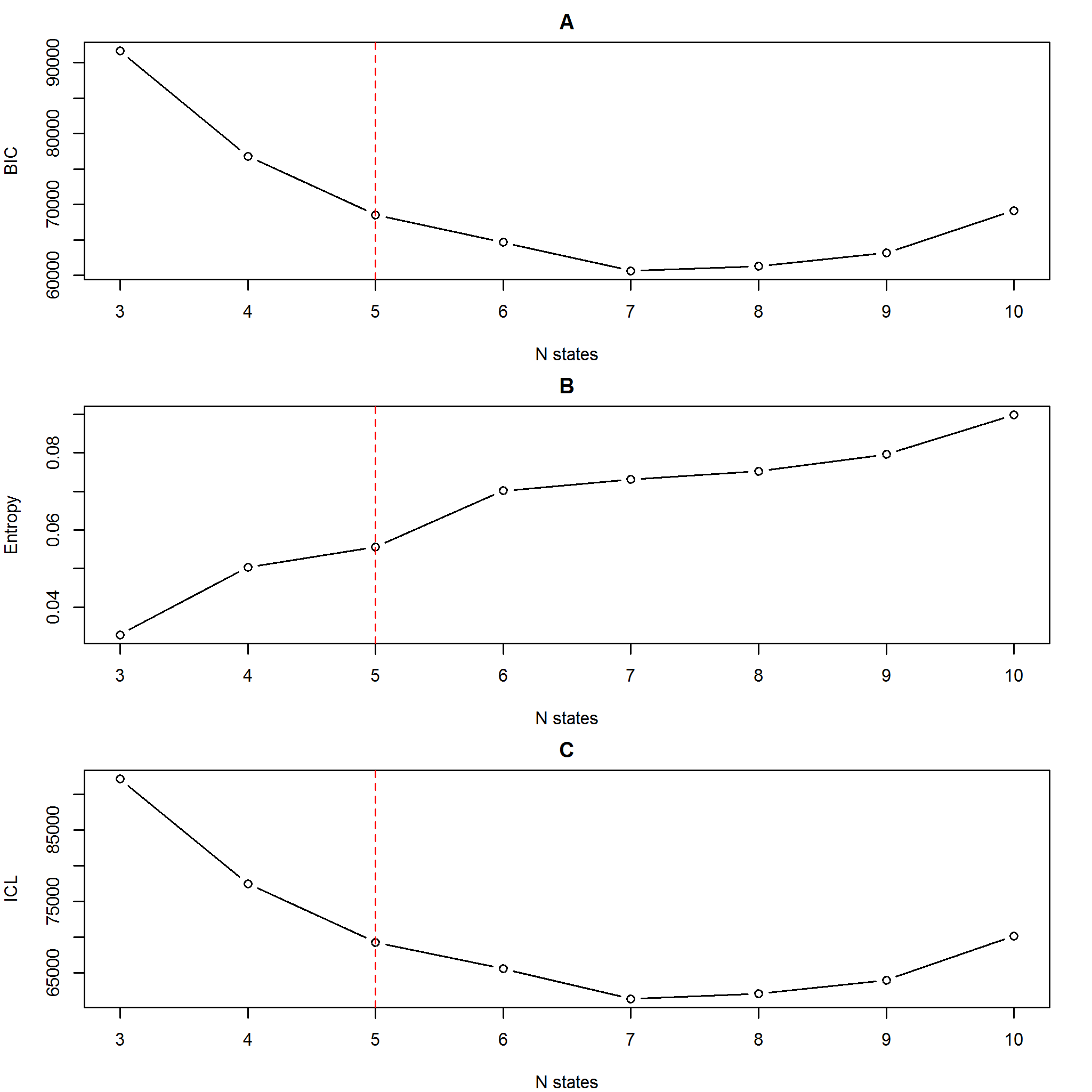
**

**Figure S5.** From a set of eight Hidden Markov Models (HMM), the best compromise HMM was selected according to selection criteria integrating model likelihood and complexity (BIC, A), model entropy which is indicative of states’ overlap (B) and the Integrated Completed Likelihood (ICL, C) which integrates both the BIC and entropy indices. We chose the optimal number of groups (between 3 and 10) by retrieving the best compromise in terms of ICL and entropy indices as well as model complexity in order to facilitate ecological interpretation. The red dotted line indicates the five-state HMM we retained.

**
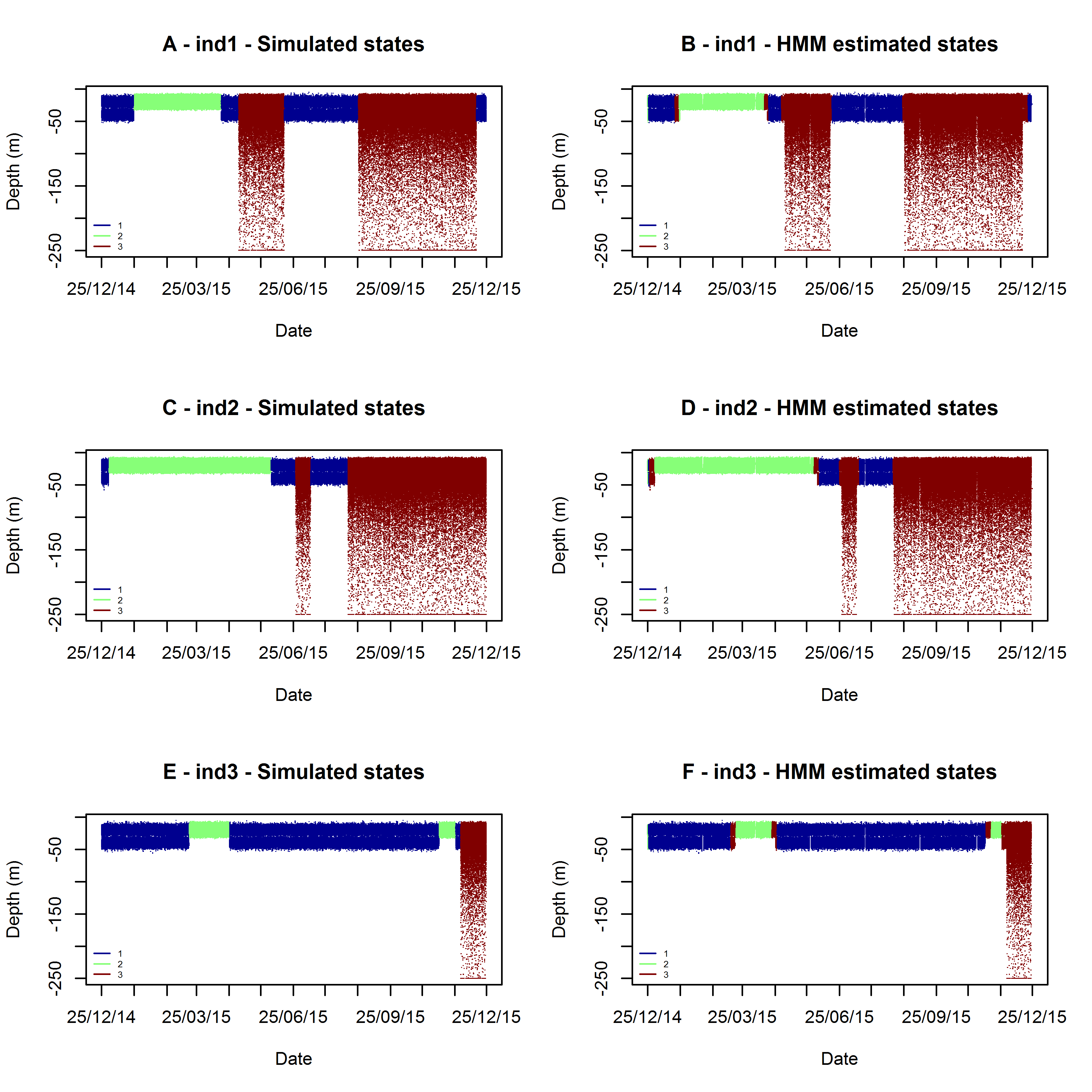
**

**Figure S6.** Known (A, C, E) and HMM estimated (B, D, F) behavioural states represented along the simulated depth time series for three individuals with different state switching dynamics (See also table S1).

**
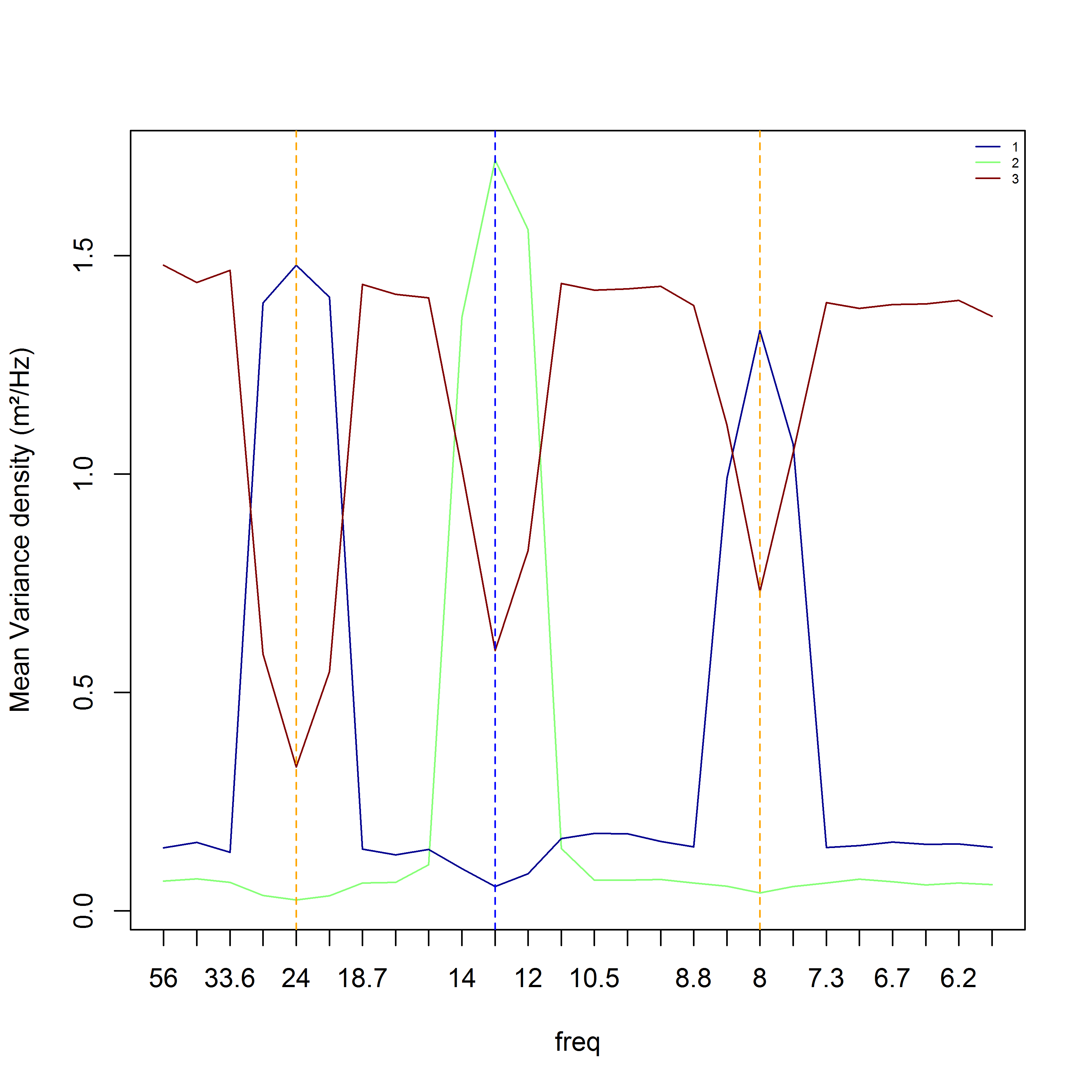
**

**Figure S7.** Spectral signature and activity levels associated to each behavioural states of the fitted three-state HMM for all individuals pooled together. The orange and blue dotted lines indicate the diurnal and tidal periodicities, respectively.
